# Remote activation of place codes by gaze in a highly visual animal

**DOI:** 10.1038/s41586-025-09101-z

**Published:** 2025-06-11

**Authors:** Hannah L. Payne, Dmitriy Aronov

**Affiliations:** 1 Zuckerman Mind Brain Behavior Institute, Columbia University; 2 Howard Hughes Medical Institute at Columbia University

## Abstract

Vision enables many animals to perform spatial reasoning from remote locations^1^. By viewing distant landmarks, animals recall spatial memories and plan future trajectories. Although these spatial functions depend on hippocampal place cells^2,3^, the relationship between place cells and active visual behavior is unknown. Here, we studied a highly visual animal, the chickadee, in a behavior that required alternating between remote visual search and spatial navigation. We leveraged the head-directed nature of avian vision to track gaze in freely moving animals. We discovered a profound link between place coding and gaze. Place cells activated not only when the chickadee was in a specific location, but also when it simply gazed at that location from a distance. Gaze coding was precisely timed by fast, ballistic head movements called “head saccades”^4,5^. On each saccadic cycle, the hippocampus switched between encoding a prediction of what the bird was about to see and a reaction to what it actually saw. The temporal structure of these responses was coordinated by subclasses of interneurons that fired at different phases of the saccade. We suggest that place and gaze coding are components of a unified process by which the hippocampus represents the location that is currently relevant to the animal. This process allows the hippocampus to implement both local and remote spatial functions.

Consider a classic example of spatial memory: an animal remembering a nut hidden at the base of a tree. Theories of hippocampal function posit that such a memory depends on place cells – in this case, the set of neurons active at the base of the tree^2,3^. Therein lies a problem: to efficiently find the tree and retrieve the nut, the animal must first recall the memory from a remote location, where a completely different set of place cells might be active. How are place cells compatible with such a remote function of the hippocampus? In visual animals, remote recall is often driven by gaze^1^. By simply looking at a place from a distance, animals can recall associated information without physically revisiting that location. Yet, it is unknown how the brain coordinates remote vision with the activity of place cells.

This problem is unsolved largely because eye tracking in freely behaving animals is extremely challenging. In addition to this technical issue, many laboratory models, including rodents, lack foveal vision^4^ and rarely orient their eyes precisely at visual targets. It is often impossible to know exactly what these animals are looking at, even when eye tracking is feasible^6–9^. In primates that have foveal vision, some hippocampal activity is correlated with gaze location^10–15^ and other visual information^16–18^. However, these animals are usually recorded stationary, or in conditions where it is hard to disambiguate gaze coding from place coding.

To address these challenges, we leveraged a unique feature of avian biology. Birds, like primates, have foveal vision and actively control their gaze to fixate visual targets^4,5,19^. However, instead of eye movements, many bird species rely primarily on head movements to shift gaze from one target to another. These head movements are much more feasible to track in a small, freely moving animal. We chose to use the black-capped chickadee – a member of a food-caching bird family that has abundant place cells in the hippocampus^20,21^. Chickadees provide a unique opportunity to simultaneously study spatial coding and gaze during unconstrained behavior.

## Head-directed gaze strategies in chickadees

**Extended Data Figure 1.**
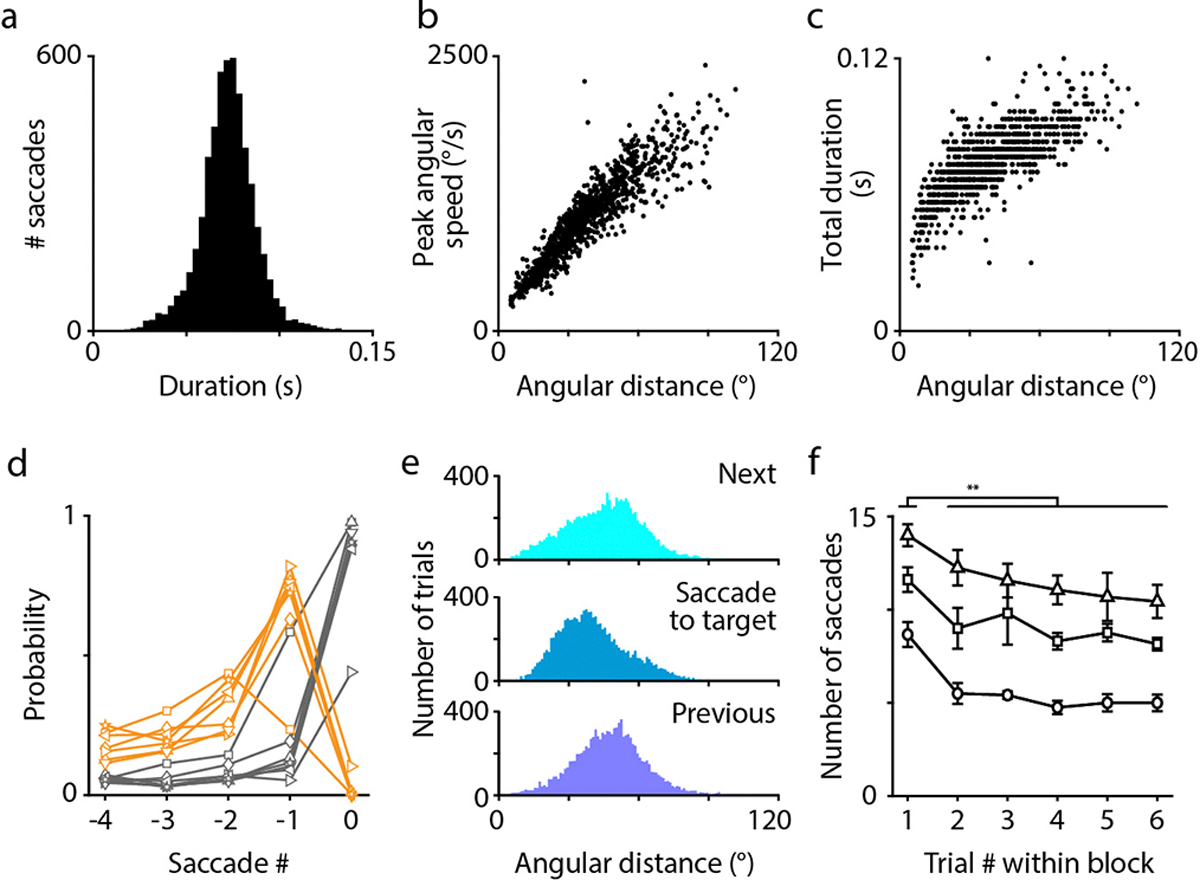
Properties of head movements in chickadees **(a)** Head saccade durations for an example session. **(b)** Relationship between angular distance travelled by the head and the peak angular speed during head saccades for the same session. **(c)** Relationship between distance travelled and saccade duration. Both (b) and (c) show strong correlations, illustrating similarities to the eye saccade main sequence in primates^62^. **(d)** Time courses of the two gaze strategies (*orange*: lateral; *black*: frontal). Each line represents one bird. Data are shown as in Fig. 1g, but for all 7 birds recorded in the X-shaped arena, averaged across sessions. **(e)** Distribution of saccade angular distances for saccades that successfully land on an unrewarded target site, as well as for the preceding and following saccades. Gazes that land on the target are produced by smaller saccades (median saccade distance 38.7° for the saccade to the target, 47.8° preceding saccade, 46.0° next saccade; saccade to target vs. previous and next, p = 0.0004 and 0.0009, two sided t-tests conducted on the median distances for each bird, n = 7 birds recorded in the X-shaped arena). This implies that gaze locations that precede successful target hits are, on average, slightly closer to the target than other gaze locations. **(f)** Number of saccades required by birds in the Blocked trial task to find the correct target. Each line represents one bird, error bars indicate mean ± S.E.M. across the medians for each session (n = 8, 13, 6 sessions for the three birds from top to bottom). Chickadees take longer to find the target on the first trial (when they have no information about which target is rewarded) than on subsequent trials (p = 0.007, 0.006, 4 × 10–8, two-sided t-test conducted separately for each bird). Note that when shifting gaze from one target to another, birds often make several intermediate saccades at other points in the environment.

**Figure 1.**
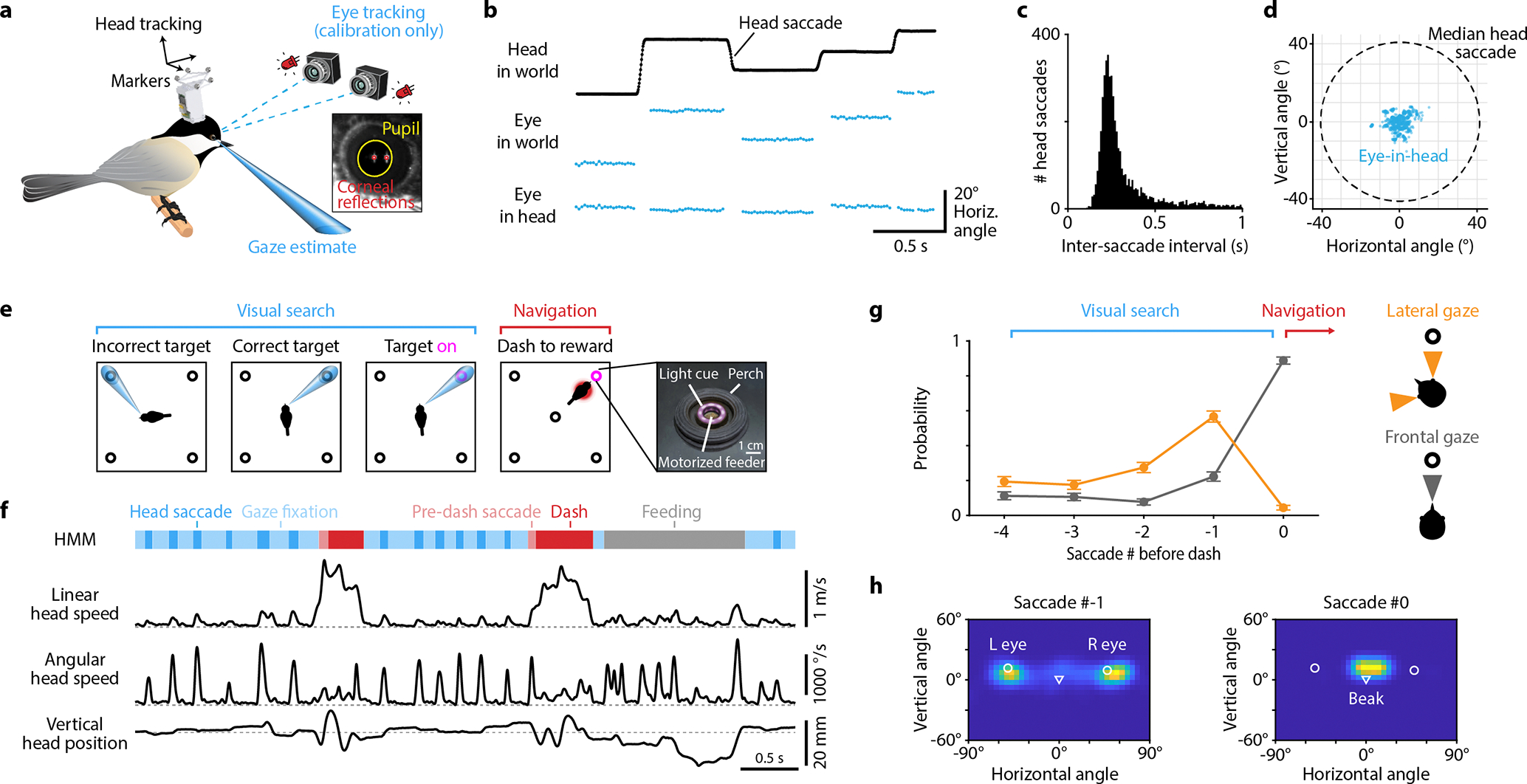
Head-directed gaze strategies in chickadees. **(a)** Head position is tracked using four infrared cameras (not shown) and reflective markers on the head. In a calibration experiment, eye position is measured using two additional cameras that track the pupil and corneal reflections of two light sources. **(b)** Head angle shows prolonged fixations interrupted by fast, ballistic movements (“head saccades”). For simplicity, only the horizontal angle is plotted. The eye could not be accurately tracked during head saccades; those points are omitted. When head position is subtracted from eye position, the residual eye position relative to the head shows very little movement. **(c)** Distribution of intervals between head saccades from a single session. **(d)** Distribution of eye positions from an example calibration experiment. Eye movements relative to the head are much smaller than the head saccades. **(e)** Schematic of the discrete visual search task, which contains a visual search period and a separate navigational period. Gaze at the correct target activates a light cue. The chickadee then navigates to that target to obtain a reward. **(f)** Different behaviors involve distinct movements of the head and can be segmented from head tracking data using a Hidden Markov Model (HMM). **(g)** Time course of the bird’s use of two different gaze strategies (lateral and frontal gaze). Saccade #0 is defined as the one immediately preceding a dash (“pre-dash saccade” in (f)). Data are mean ± standard error of the binomial proportion for one example session (n = 250 dashes). **(h)** Distribution of head orientations relative to the target, averaged across all #0 and #−1 saccades for the same bird as in (g). Symbols mark the orientation of the two pupil centers and the tip of the beak. Saccade #0 was usually frontal (using both eyes). Prior to that, saccades tended to be lateral (using one eye at a time).

All birds use their heads to direct gaze, but the nature of head movements and the extent of additional eye movements are highly variable across species^4,22^. We therefore started by characterizing these behaviors in chickadees. For head tracking, we adapted a multi-camera system^23^ that triangulated infrared-reflective markers on the bird’s head (Fig. 1a). As in other species^4,5^, these measurements revealed a saccadic-like behavior (Fig. 1b). Chickadees produced fast, ballistic changes in head angle (“head saccades”) interleaved with periods of stable head angle (“gaze fixations”). Head saccades were 76 ± 21 ms in duration, occurred at instantaneous rates of 3.8 ± 1.4 Hz, and were in several additional ways remarkably similar to eye saccades in primates^1^ (mean ± standard deviation, n = 3.3×10^5^ saccades in 8 birds; Fig. 1c, Extended Data Fig. 1a–c).

**Extended Data Figure 2.**
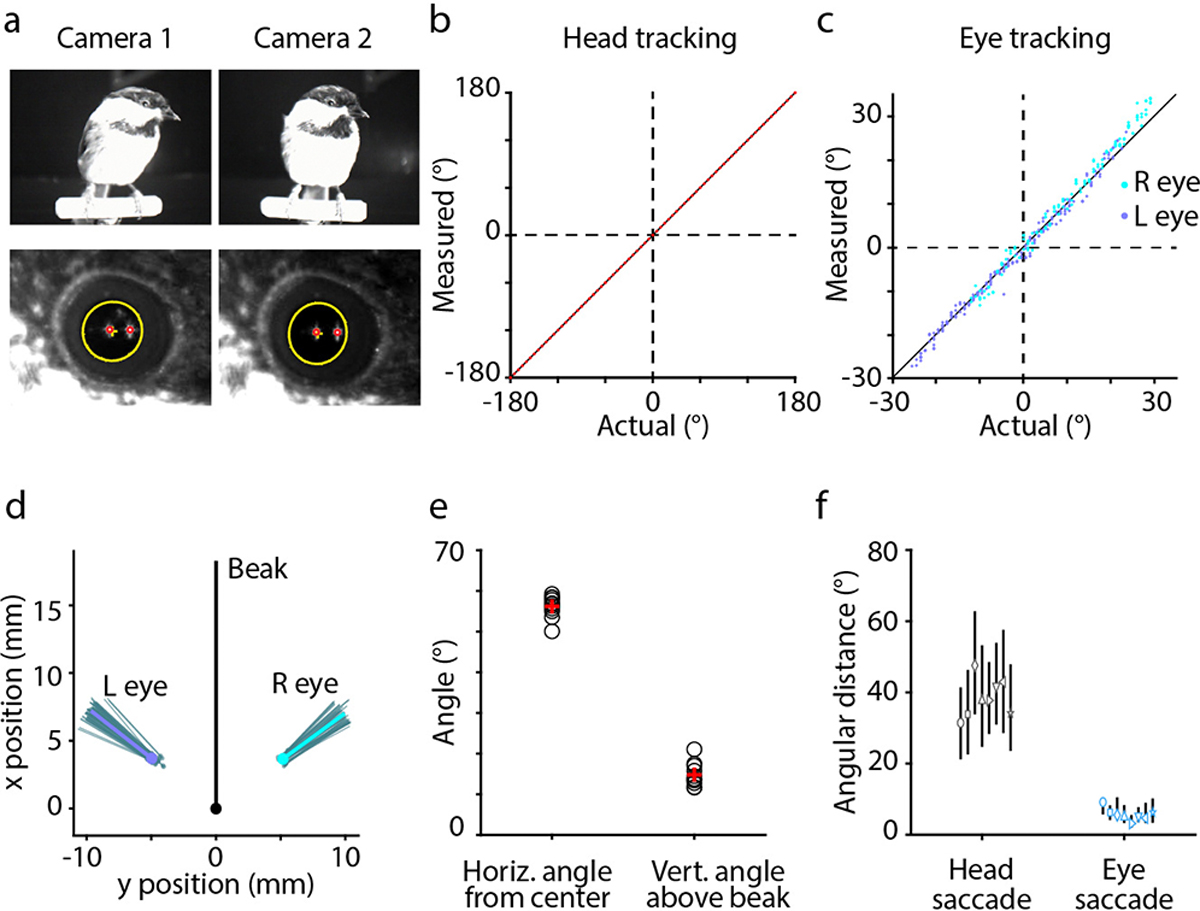
Eye tracking method and properties of eye movements in chickadees **(a)** Video-oculography using two infrared light sources. The chickadee is perched close to two cameras, which acquire video frames from slightly different angles (*top*). The pupil (yellow) and the corneal reflections of the two light sources (*red*) are detected in the videos. Head tracking is conducted simultaneously using an array of infrared reflective markers attached to the bird’s implant (*not shown*). **(b)** Accuracy of head tracking: 0.046° root-mean-squared error (RMSE). *Red dots*: measurement; *Black line*: unity line. **(c)** Accuracy of eye tracking: 1.78° RMSE measured across fixations, 1.79° measured across individual frames. *Dots*: individual fixations. **(d)** Position of the eye (*dots*) and the orientation of the optical axis (*lines*) relative to the head. Projections onto the horizontal axis are shown. *Gray*: individual video frames for a single calibration experiment. *Blue*: average across frames. **(e)** Orientation of the optical axis across all birds (*black symbols*) and the average across birds (*red symbols*). **(f)** Angular displacement of the head and the eye during head saccades. Symbols indicate medians for each bird; lines indicate 25th and 75th percentiles (n = 4341 – 144521 head saccades per bird, n = 8 – 55 eye saccades per bird).

We then performed a calibration experiment in which we tracked eye movements in addition to head movements. For this purpose, we engineered a dual-camera video-oculography system that estimated the pupillary axis using corneal reflections of two infrared light sources (Fig. 1a, Extended Data Fig. 2). These measurements were possible only when we encouraged chickadees to perch directly in front of the cameras. We found very little movement of the eyes relative to the head: 5.4 ± 0.4° median absolute deviation, nearly an order of magnitude smaller than the movement of the head itself (n = 8 birds, Fig. 1b,d, Extended Data Fig. 2f). We concluded that chickadees almost exclusively rely on head saccades to direct gaze during free motion. Head tracking is therefore sufficient to determine where a bird is looking, provided that gaze targets in a behavioral task are separated by more than ~10°. Unlike eye tracking, which requires the head to be nearly stationary relative to the camera, head tracking is feasible in chickadees freely navigating across a behavioral arena.

To study place and gaze coding, we trained birds on a discrete visual search task (Fig. 1e; Supplementary Video 1). The chickadee visited five visually identical sites: one at the center and four near the corners of a 61-cm arena. Each site consisted of a perch, a light cue, and a motorized feeder. On each trial, one of the four corner sites was randomly chosen as the reward location. The chickadee started the trial by perching at the central site. The rewarded site was then indicated by a light. Once the light turned on, the bird could approach the indicated site to retrieve a piece of a sunflower seed, then return to the center to initiate a new trial. Chickadees typically approached rewarded sites via fast, direct movements that we call “dashes”. Although the arena was two-dimensional, these dashes typically covered only the X-shaped region that connected the center with the corner sites. We used a Hidden Markov Model to segment dashes, gaze fixations, saccades, and feeding periods in the head tracking data (Fig. 1f). Chickadees performed 163–304 trials (25th-75th percentile, n = 58 sessions across both tasks described below), with each site rewarded on roughly 1/4 of the trials in any given session.

We first ran a simple version of the task to characterize the chickadees’ visual search behaviors. In this version, the light cue that indicated the rewarded site turned on after a random delay (up to 5 s) from the start of the trial. We found that chickadees used two distinct gaze strategies in our task (Fig. 1g–h, Extended Data Fig. 1d). During the delay period, they shifted gaze between different sites using one eye at a time (“lateral gaze”). In other words, chickadees oriented their head to align either the left or the right pupil with one of the sites. Because the light cue was intentionally dim, chickadees probably used this behavior to search for the rewarded site using the foveal region of the retina^19^. After locating the light, birds instead oriented the beak toward the target (“frontal gaze”), viewing the rewarded site with the non-foveal region of both eyes. They usually followed this frontal gaze by a dash. Other bird species use similar strategies, relying on lateral gaze to investigate objects and frontal gaze during directed movements^24–26^.

**Extended Data Figure 3.**
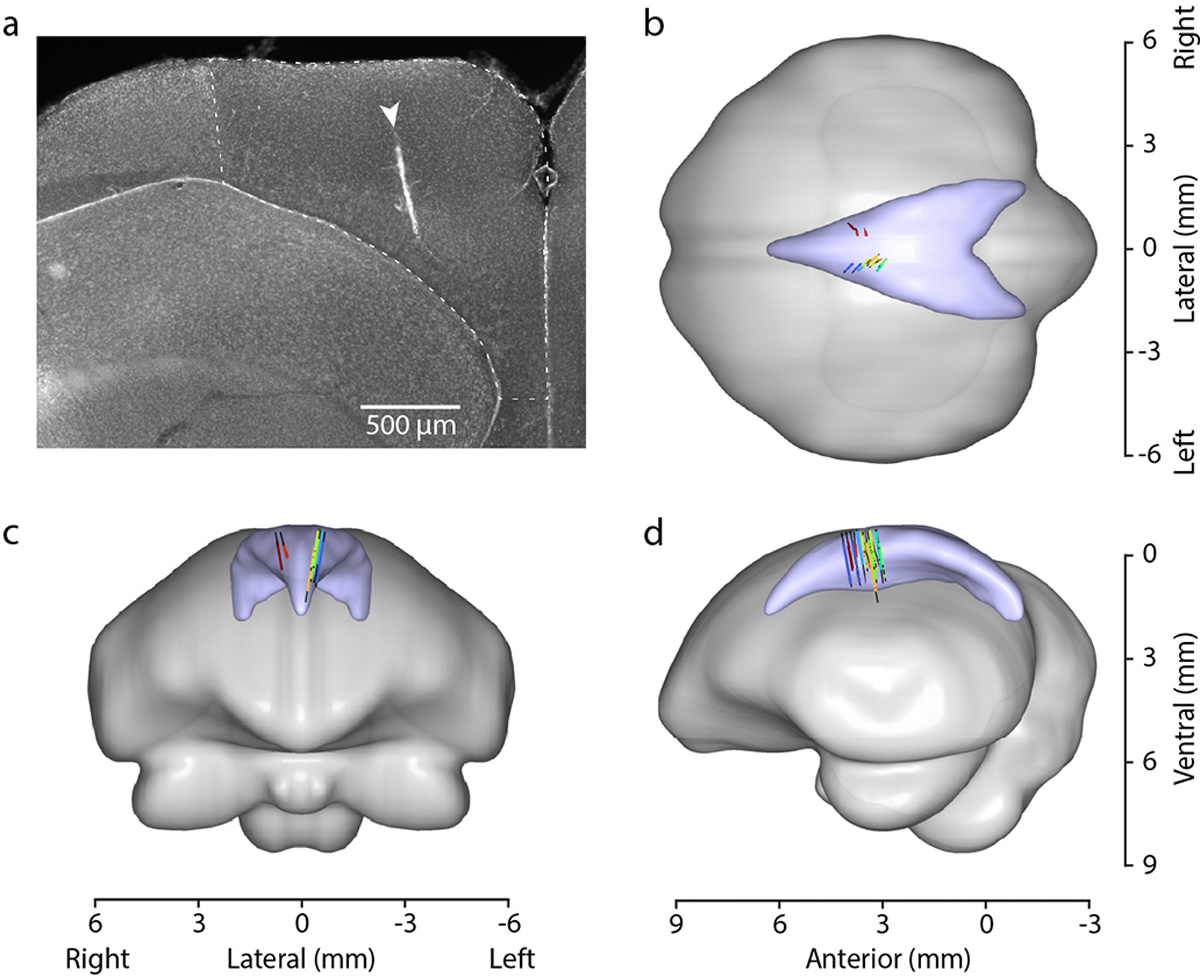
Histological analysis of recording locations **(a)** Coronal section of a typical recording location in the hippocampus, showing the track of the recording probe. The section is labeled with DAPI, which clearly delineates the lower-density hippocampus from the higher-density dorsolateral region (DL) that is directly lateral to the hippocampus. **(b-d)** Locations of all recorded cells registered to a 3D model of the chickadee brain constructed using data from^63^. *Colored symbols:* cells included in the paper, *black lines:* electrode tracks. Track locations were confirmed histologically for 8/14 probe tracks, yielding 2504/2658 cells in the X-shaped tasks and 351/361 cells in the all-to-all task (note that 5/9 birds were implanted with two-shank silicon probes). The locations of the remaining cells were estimated from stereotaxic coordinates. Lambda is located at 0 mm in the displayed coordinate axes.

With these results in mind, we modified the task to create a closed-loop version. Instead of enforcing a delay period, we activated the light cue in response to the bird gazing at the correct site. Because chickadees had no *a priori* knowledge of which site was rewarded, they often gazed at multiple incorrect sites before choosing the correct one. The closed-loop structure allowed us to precisely control the timing of the visual stimulus relative to the saccade. It also ensured that the cue was not detectable by peripheral vision during off-target saccades; this feature will become important later. In this closed-loop task, we performed recordings of the anterior hippocampus (Extended Data Fig. 3) using silicon probes.

## Place cells are activated by remote gaze

**Figure 2.**
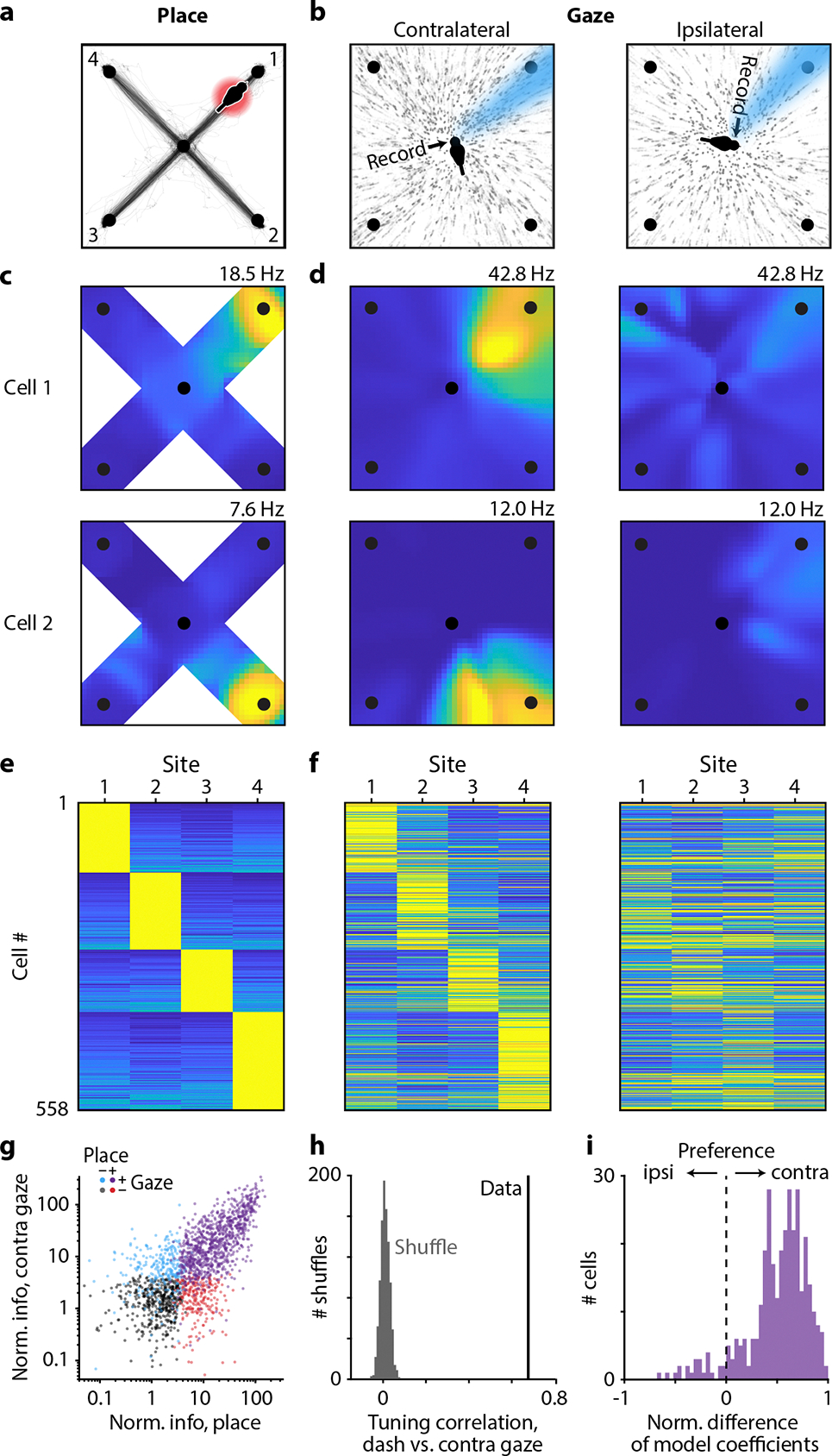
Place cells are activated by remote gaze. **(a)** Place representation is measured during times when the bird is dashing toward an outer target. Black trace: trajectory of the bird during such periods in a single example session. This trajectory is mostly confined to the X-shaped portion of the arena that connects the target sites (1–4) to the center. **(b)** Gaze representation is measured during times when the bird is saccading from the center of the arena. Black dots: projected gaze in the same example session, plotted separately for the eyes contralateral and ipsilateral to the hippocampal recording. **(c)** Mean firing rate as a function of the bird’s location for two example place cells. Only locations on the X-shaped part of the arena are shown because the chickadee almost never visits other locations. Color scale is from 0 (blue) to the indicated maximum (yellow). **(d)** Mean firing rate as a function of gaze location for the same two cells. **(e)** Peak firing rate during dashes to each of the four target sites. Excitatory place cells with strong place selectivity for one of the targets (>0.5 selectivity index) are shown. Cells are sorted first by location of their strongest response and then by the magnitude of their second-strongest response. Each row is separately normalized from 0 (blue) to the maximum (yellow). **(f)** Coefficients of a model that fits gaze responses as a combination of tuning to contralateral and ipsilateral gaze. Cells and sorting are the same as in (e). Rows are normalized to the maximum across both matrices in (f). Note that cells are not excluded from this plot based on their gaze responses; the relationship between place and gaze tuning is shown across the entire population of place cells. **(g)** Strength of place and gaze tuning across all excitatory cells. Cells are colored based on a statistical threshold for place and/or gaze tuning; these colors are not meant to represent separable classes of neurons. “Normalized information” is mutual information between spikes and the behavioral variable (place or contralateral gaze, discretized into four target sites), divided by the mean for shuffled data. The two types of tuning are strongly correlated. **(h)** Correlation of tuning curves for place and contralateral gaze (r = 0.68, n = 558 cells). Tuning curves are measured across the four targets for all place cells shown in (e,f). Shuffled distribution is obtained by scrambling cell identities. **(i)** Comparison of contralateral and ipsilateral gaze responses, using model coefficients in (f) for each cell’s preferred dash target. Included cells are those in (h) with >0.5 selectivity both for place and for gaze with at least one of the eyes (n = 289 cells). Normalized difference is (c-i)/(c+i), where c and i are contralateral and ipsilateral coefficients. Gaze responses are almost entirely explained by contralateral tuning.

We measured place tuning and gaze tuning in the firing of hippocampal neurons. To match published work^27,20,21^, we analyzed place tuning only during periods when the chickadee was locomoting – i.e., dashing between sites (Fig. 2a). In contrast, we analyzed gaze tuning during stationary visual search periods, when the bird was saccading and fixating at targets (Fig. 2b). We started by examining activity as a function of contralateral gaze – i.e., the gaze target of the eye contralateral to our hippocampal recording. To quantify place tuning and gaze tuning, respectively, we measured firing rates during dashes and during gazes at each of the four target sites. We then computed mutual information between firing rate and site identity. As in other behavioral tasks, many hippocampal neurons in chickadees were place-tuned: of the 1929 putative excitatory cells in seven birds, 62% were classified as place cells (p < 0.01, mutual information compared to shuffled data). We found that a similarly large fraction of cells were gaze-tuned: 57% of the same 1929 neurons (p < 0.01). Many of these cells had strong gaze fields that were tightly localized in the environment, qualitatively similar to conventional place fields (Fig. 2c–d).

There are other known situations in which the hippocampus encodes more than one experimental variable^3,18,20,28,29^. In these cases, different kinds of selectivity are usually mixed randomly within the recorded population. To our surprise, this was not true for place and gaze tuning. The amounts of information encoded about place and about gaze were strongly correlated across cells (Fig. 2e–g). In fact, 75% of all place cells were also significantly tuned to gaze, compared to 57% expected from random mixing.

Not only did the same neurons encode place and gaze, but these two representations had a striking overlap in space (Fig. 2c,d). To quantify this overlap, we selected place cells that had a strong preference for dashes toward a single site. Tuning curves for place and gaze were strongly correlated in these cells (Fig. 2e,f,h). In those cells that also had a strong preference for a single gaze target, the preferred place was the same as the preferred gaze in 95% of cases, compared to ~25% expected by chance. These analyses show that place and gaze tuning are not represented independently in the hippocampus. Rather, remote gaze activates place representations. In other words, a place cell is active not only when a bird is physically in a certain location, but also when the bird simply looks at that location from a distance.

In many behaviors, the hippocampus can represent the future location of the animal^30,31^. In our task, birds often look at sites before visiting them. Therefore, a conceivable explanation of our results is that the hippocampus actually represents future location, which happens to correlate with gaze. To determine whether gaze responses were specifically related to visual behavior, we relied on a unique feature of avian anatomy. As in mammals, the avian hippocampus receives input from multiple visual pathways^32–34^. However, in most birds, the optic tract fully crosses to the contralateral hemisphere at the optic chiasm^35^. There is also very limited cross-hemispheric communication in the visual system due to the lack of a corpus callosum. As a result, visual functions are highly lateralized^36,37^. If gaze signals are actually driven by future location, we should observe them bilaterally, but if they are specific to gaze, we might expect them to be lateralized in the hippocampus.

**Extended Data Figure 4.**
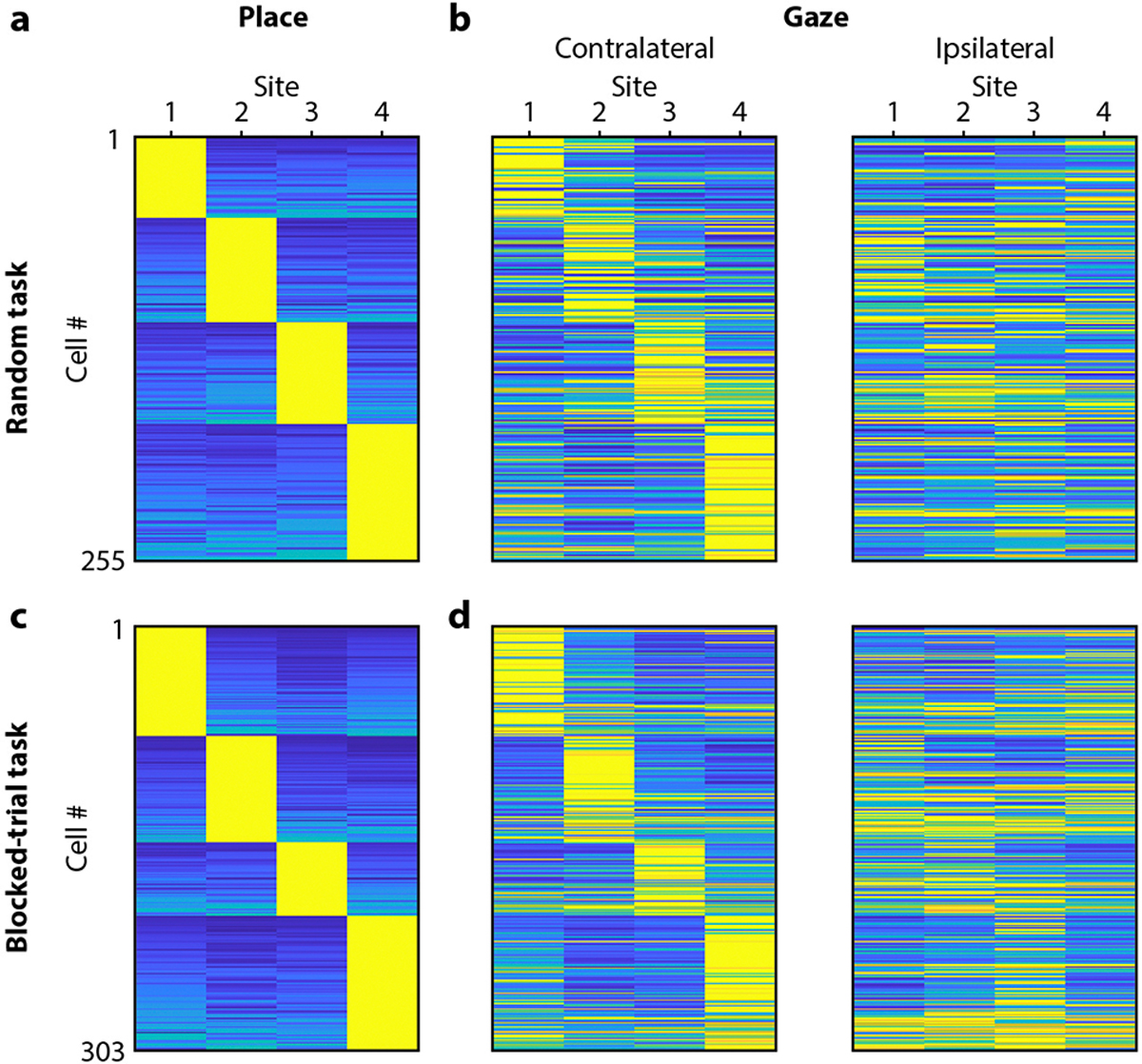
Comparison of place and gaze coding across tasks **(a-b)** Place and gaze coding, shown as in Fig. 2e–f, but only for cells recorded in the Random task, where the location of the rewarded target was chosen randomly on each trial. Each row is separately normalized from 0 (blue) to the maximum (yellow). **(c-d)** Same, but only for cells recorded in the Blocked-trial task, where the same rewarded target was repeated for six trials in a row.

We found place and gaze tuning in both hemispheres. However, gaze tuning was evident only when we analyzed the eye contralateral to the recorded hippocampus (Fig. 2d). Neurons responded only when the contralateral, but not the ipsilateral, eye looked at the preferred target. To illustrate this result across the population, we implemented a model that fit neural activity as a combination of tuning to ipsilateral and contralateral gaze. Such a model was necessary because chickadees sometimes gazed simultaneously at two sites with different eyes. In this model, activity was almost entirely explained by contralateral gaze (Fig. 2f,i). This result was true regardless of whether birds were allowed to use either eye or only the contralateral eye to trigger the reward (Extended Data Fig. 4). We concluded that gaze tuning is truly specific to where the bird is looking.

**Extended Data Figure 5.**
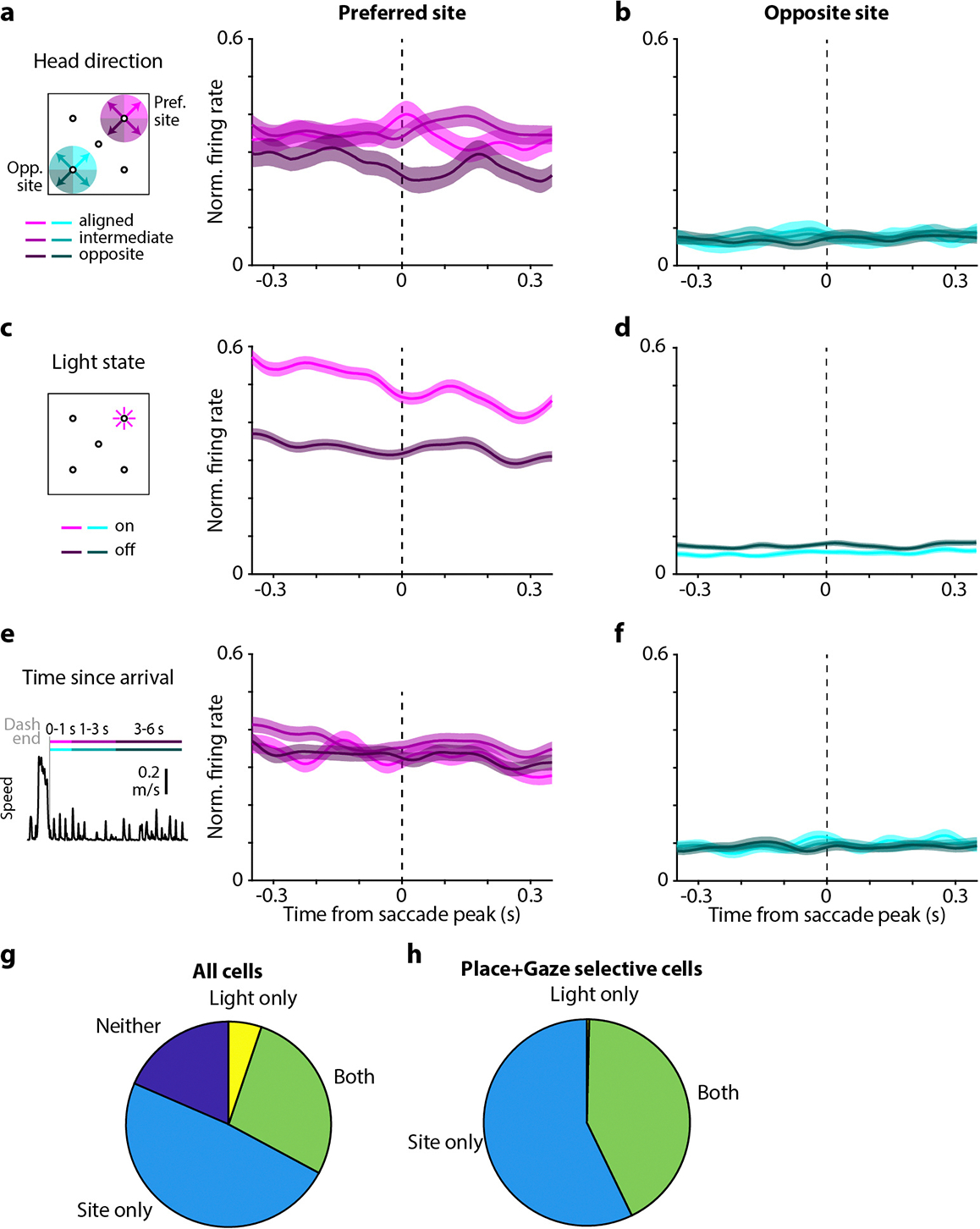
Place coding is not explained by simple visual responses **(a-b)** Saccade-aligned firing rates averaged across cells, when the bird is located at one of the corner target sites, but gazing elsewhere in the environment. Included are cells with place and gaze selectivity (>0.5) for one of the sites. For each cell, firing rates are plotted for trials when the bird is at that cell’s preferred site and when the bird is at the opposite site. Saccades are grouped according to the azimuthal direction the bird is facing. To eliminate saccades in which the bird is gazing down at the feeder, saccades are only included if the elevation of gaze is within ±30° elevation from the average vector of gaze at targets within each session (average elevation –9° across sessions). The direction of gaze is given by the contralateral eye. In all cases, cells remain selective for the bird’s location at their preferred site. Error bars indicate mean ± S.E.M. across cells. **(c-d)** Same as (a-b), but saccades are grouped according to whether the indicator light is on or already turned off. Although cells respond more strongly when the light is on at their preferred site, this response does not explain their site preference. **(e-f)** Same as (a-b), but saccades are grouped according to the time after the bird’s arrival at the target site. Elapsed time does not explain the site preference. **(g)** Results of a generalized linear model that evaluated each cell’s tuning for gaze location and the state of the viewed light cue (on or off). For each saccade, we counted spikes from −100 to +300 ms, and for each cell used the MATLAB function fitglme with a log link function and Poisson distribution. Very few cells (5.2%, 131/2524 with a statistical threshold of p = 0.05, two-sided log-likelihood ratio test) were tuned to the indicator light without also being location-tuned. **(h)** Same as (g), but for the 278 cells that had place and gaze selectivity of >0.5. Only one cell was significantly light-tuned without also being location-tuned.

**Extended Data Figure 6.**
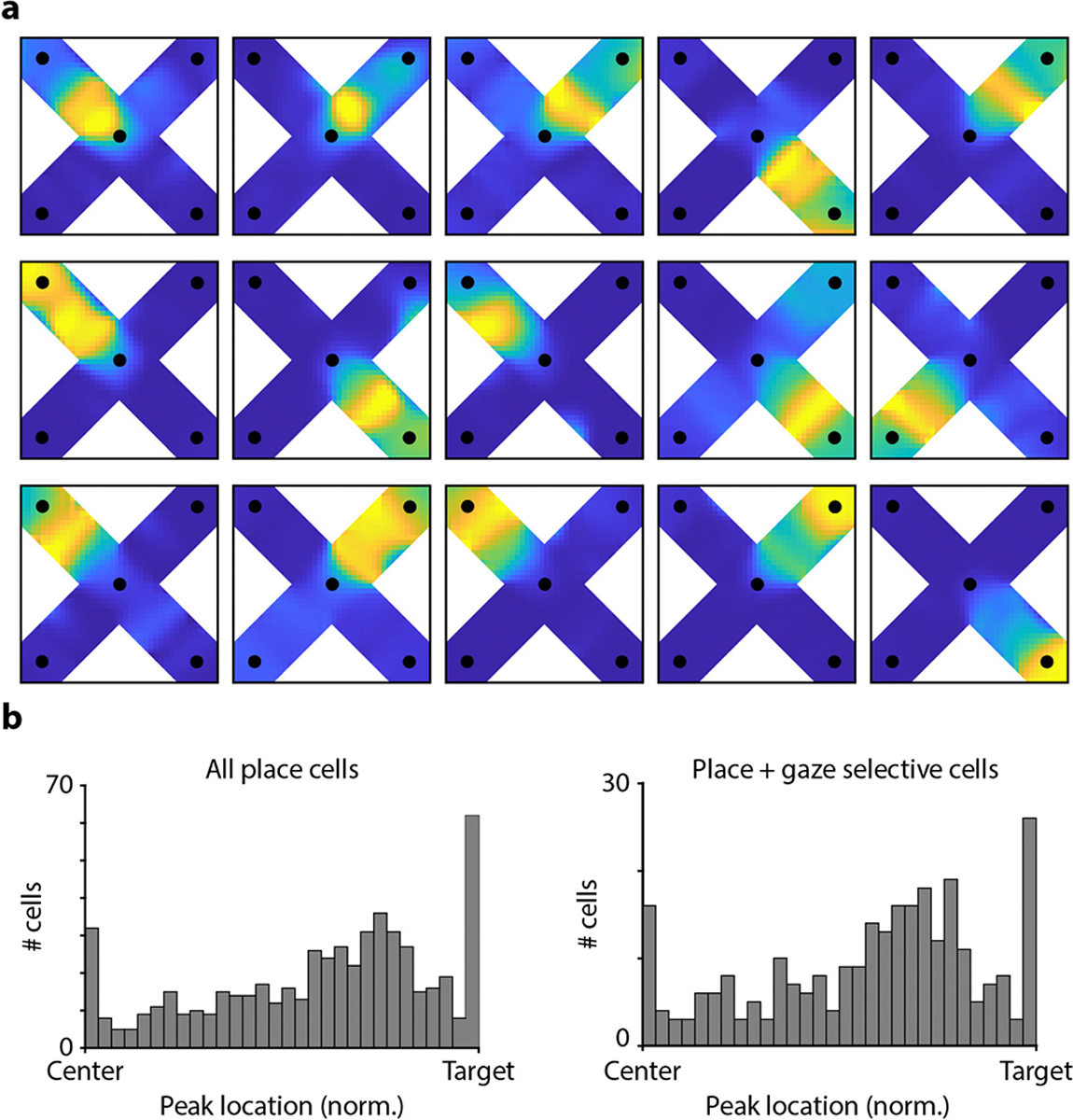
Distribution of place fields in the arena **(a)** Example place maps for neurons that had firing fields at various locations along the path of the bird. In order to match the conventional way place cells are analyzed in the literature, spikes are not shifted by the optimal time lag for each neuron. Otherwise, the maps are plotted as in Fig. 1c. **(b)** Distribution of place field locations for all place cells (*left*) and only cells with place and gaze selectivity >0.5 (*right*). Place field location was defined as the location of the maximum firing rate for each cell. The first and last bin of the histogram are over-represented because the maximum was measured on a truncated segment of the arena between the central site and the target. Although more cells have place fields closer to the target than to the center, there are many cells with place fields far from the target that have significant gaze coding (*right*).

Are responses during dashes truly tuned to the bird’s location? Because place and gaze tuning overlap, an alternative is that apparent place coding during dashes can instead be explained by visual responses to the target sites. We considered this unlikely. First, all four targets were visually identical. Spatially selective gaze responses therefore had to depend on the spatial arrangement of distant landmarks, not only the local features of the target sites. Second, targets looked very different to birds during gazes and during dashes, yet produced similar responses. During gazes, targets were viewed by the foveal part of the retina and appeared small due to the large distance from the bird. During dashes, they were viewed by a non-foveal part of the retina and appeared several times larger. To further rule out visual responses, we analyzed periods of time when the chickadee had arrived at the target site but was no longer looking at it. Cells retained their place selectivity during these times, regardless of which way the bird was facing and whether the light cue was turned on or off (Extended Data Fig. 5a–f). We also found that very few cells responded to the light cue without regard for its location (Extended Data Fig. 5g–h). Finally, we identified many cells with place fields along the paths to the targets, rather than directly at the targets (Extended Data Fig. 6). We concluded that chickadee hippocampal activity is truly spatial, and cannot be explained by visual responses to target sites or the reward indicator light.

**Extended Data Figure 7.**
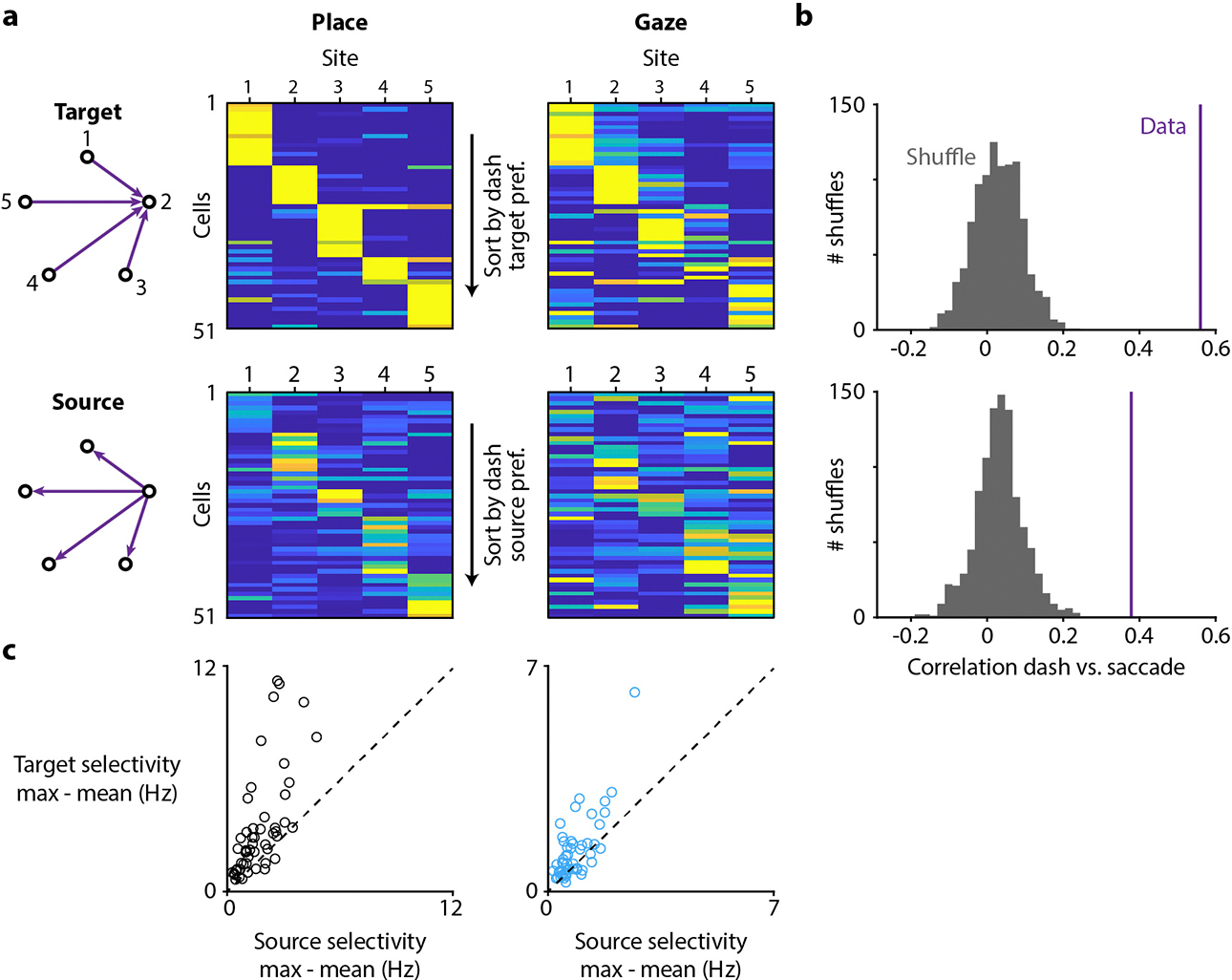
All-to-all task to disambiguate gaze target from the bird’s location **(a)** Activity in the All-to-all task, where the chickadee was located at one of five sites and gazing at one of the other sites. After this visual search period, the chickadee dashed directly from one site to another. The location of the bird prior to the dash was the “source”, while the target of gaze and the endpoint of the dash was the “target”. Activity of each cell is shown during dashes (“Place”, *left*) and during gaze fixations (“Gaze”, *right*). Firing rates are calculated as a function of either the target site (*top*) or the source site (*bottom*). Included are cells with place and gaze selectivity (>0.67), either for the target or the source, and with baseline-subtracted response > 0.5 Hz. Each row is normalized from 0 (*blue*) to maximum (*yellow*) across target and source measurements, separately for place and gaze. **(b)** Correlation of the tuning curves for place and gaze (i.e. the rows of the matrices in (a)). *Top*: correlation of target tuning curves; *bottom*: correlation of source tuning curves. In both cases, correlations for actual data are higher than for a shuffled distribution, in which cell identities were scrambled. **(c)** Comparison of selectivity for target and for source. Selectivity for each neuron was measured by subtracting the mean of the five values in its tuning curve from the maximum of those five values. For both place (*left*) and gaze (*right*), selectivity is higher for the target than for the source.

Another consideration is that in the “X-shaped” task presented so far, chickadees always performed the visual search from the same central location of the arena. Could hippocampal activity encode the direction of gaze, rather than the location of the gaze target? We could not test this possibility in the X-shaped task because chickadees rarely gazed at any site when they were not perched at the center. We therefore trained three birds on a separate “all-to-all” task, in which they dashed directly between five outer target sites without returning to a central perch (Extended Data Fig. 7). We found that gaze responses depended on both the site where the chickadee was located (the “source”) and the site at which it was looking (the “target”). Responses for the same target from different sources were more similar than responses for different targets from the same source. We concluded that gaze responses predominantly encoded the location of the visual target, even though they were also partially affected by the location of the bird. These responses could not be explained by the direction of gaze alone.

## Gaze responses encode an internal prediction

**Figure 3.**
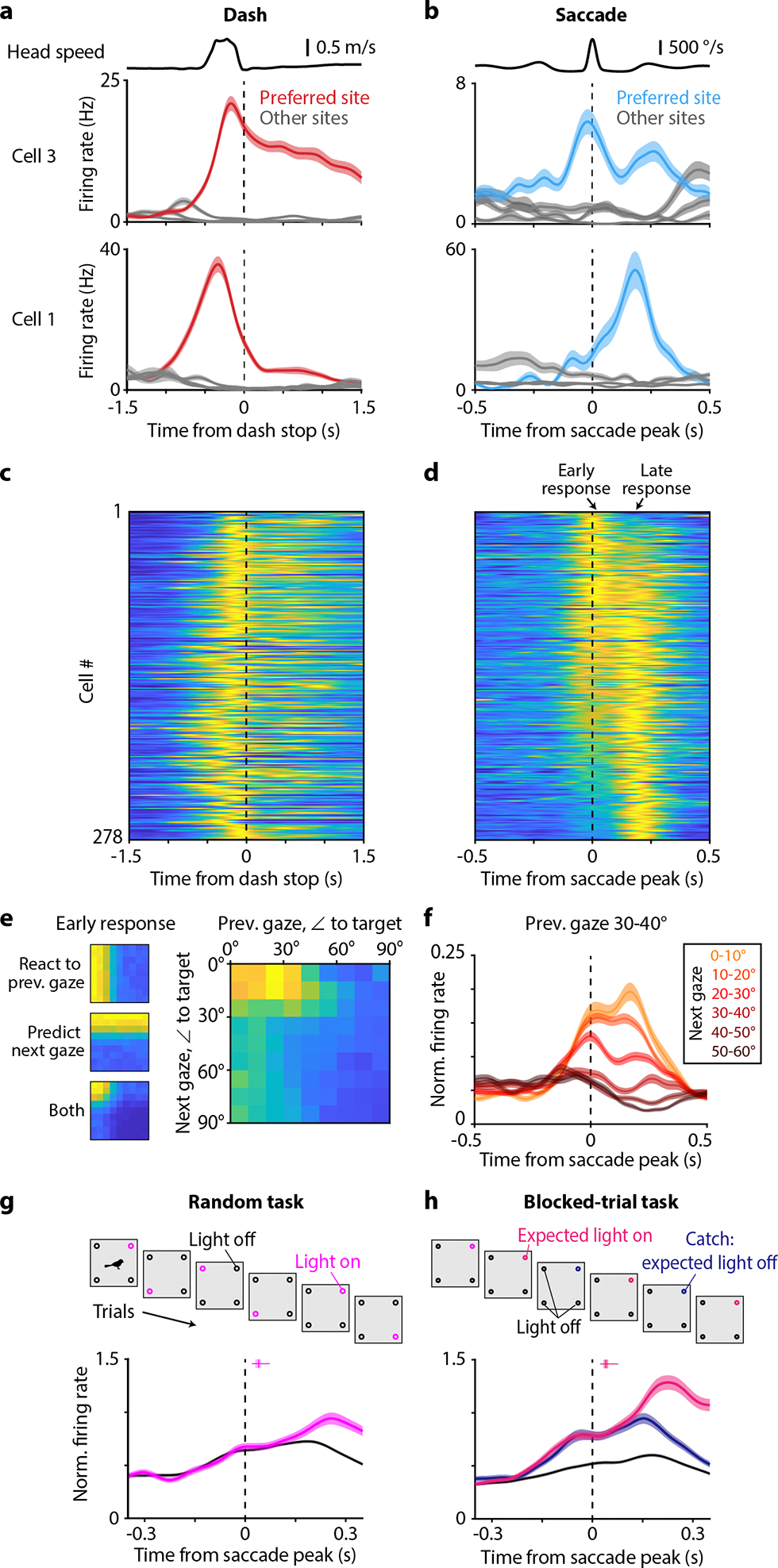
Gaze responses encode an internal prediction. **(a)** Activity of two excitatory cells aligned to dashes toward each of the four targets. Traces are mean ± S.E.M. across dashes. Average linear speed of the head across the entire dataset is shown above. **(b)** Activity of the same two cells aligned to saccades toward the same four targets. Traces are mean ± S.E.M. across saccades. Average angular speed of the head is shown above. **(c)** Activity of cells aligned to dashes toward their preferred target. Included are excitatory cells that have strong selectivity (>0.5) of both place and gaze responses for the same target. Each cell’s activity is normalized from 0 (blue) to its maximum (yellow). **(d)** Activity of the same cells aligned to saccades toward their preferred target. Cells are sorted by the difference in firing during the early and the late phases of the response (centered on 17 and 187 ms); the same sorting is applied to (c). **(e)** Amplitude of the early response (at 17 ms relative to a saccade) in the same cells as in (c-d), as a bivariate function of the distances to the target of the gazes that preceded and followed that saccade. *Left:* three hypotheses for what the bivariate function may look like if the early response is purely a reaction to the previous gaze, purely a prediction of the next gaze, or a function of both. *Right:* actual data show that the early response is a function of both. Data are normalized firing rates averaged across neurons, from 0 (blue) to 0.17 (yellow). **(f)** Responses to all saccades that were preceded by a gaze 30–40° from the target, grouped by how far the next gaze landed from the target. These saccades correspond to the 4^th^ column of the matrix in (e). **(g)** Responses to the preferred gaze target in the Random task, separately for saccades when that target was correct (indicated by the light turning on) vs. incorrect (indicated by the light staying off). Included are excitatory neurons with significant place and contralateral gaze tuning, peak firing rate of >1 Hz in the Light-off condition, and at least 5 saccades to the preferred gaze target (n = 271 cells); each cell’s activity is normalized to the peak rate during saccades in the Light-off condition. Box and whisker: 5^th^, 25^th^, 50^th^, 75^th^, and 95^th^ percentiles of the latency to the light turning on (median = 40 ms). **(h)** Responses to the preferred gaze target in the Blocked-trial task, separately for saccades when the chickadee expected the target to turn on vs. stay off (n = 402 cells; median latency to light on 40 ms). The former category includes catch trials, when the target was expected to turn on, but stayed off. In (f-h), traces are mean ± S.E.M, averaged within a cell and then across cells.

An overarching question of our study is whether the hippocampus can recall internal information about the visual world. For this purpose, it is not sufficient to simply react to visual stimuli; rather, neural responses should contain additional, internally-driven information about gaze targets. To test this idea, we first asked whether the timing of gaze activity was consistent with a purely sensory response. We aligned neural activity to the time of peak angular head velocity during each saccade, separately for each target site. We compared these responses to the timing of activity during dashes. Surprisingly, we found that saccade-aligned activity, but not dash-aligned activity, was biphasic (Fig. 3a–d). Neurons produced the first peak in firing (“early response”) during the saccade itself at 17 ± 14 ms, and then the second peak (“late response”) at 187 ms ± 4 ms (n = 278 place- and gaze-selective cells, mean ± bootstrapped standard error; Fig. 1c). Note that the “early” response relative to one saccade coincides in time with the “late” response relative to the previous saccade. Most neurons participated in both phases of the response, though the relative amplitudes of the two peaks varied across cells. The late response was compatible with latencies expected in the avian visual system^38^. In contrast, the early response occurred largely before the chickadee fixated on the preferred target and started even before the head began to move.

**Extended Data Figure 8.**
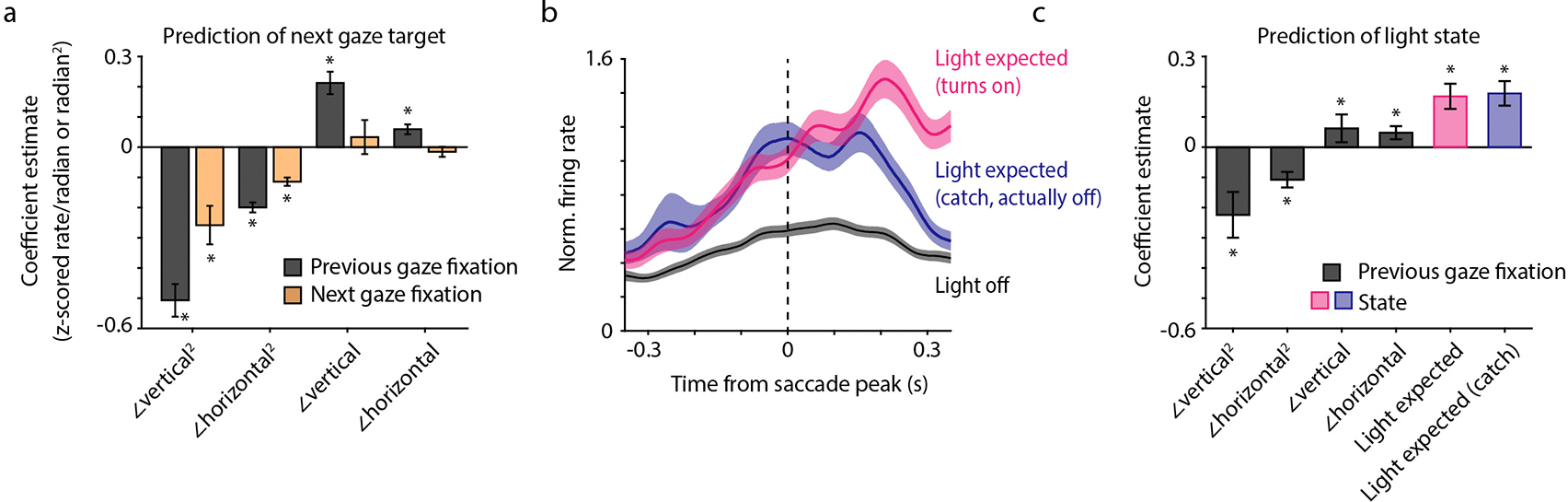
Early peak during gaze fixations partly predicts the upcoming gaze **(a)** Coefficients of a linear mixed effects model that fit the early response (at +17 ms relative to saccade peak) as a function of eight behavioral variables (fixed effects), and allowed the intercept to vary for each cell (random effect). Four of the variables accounted for the gaze fixation preceding the saccade, and the other four accounted for the gaze fixation following the saccade. For the previous and next fixation, variables included the horizontal and vertical deviations from the target (which could be positive or negative), as well as the squared values of these deviations. Included are cells with place and gaze selectivity >0.5 (n = 278 cells). Asterisks indicate significant coefficients (p < 10^−9^ for all, p-value for the t-statistic of the hypothesis that the corresponding coefficient is different from zero, returned by MATLAB function fitlme. We then adjusted each p-value for multiple comparisons using a Bonferroni correction). Error bars indicate 95% confidence intervals for the coefficient estimates. The key conclusion is that the early response depended on the location of the next fixation. **(b)** Same as Fig. 3h, but only including trials where the previous fixation was 30–40° from the preferred target. Note that the early response still depends on the bird’s expectation of the light turning on, even though the previous fixation is clamped in the same narrow range of angles for all conditions. Due to the small subset of trials, the number of included cells is now smaller than in Fig. 3h (n = 387, 377, and 364 cells for the *black*, *blue*, and *pink* traces). **(c)** Coefficients of a model that fit the early response in the Blocked-trial task and included variables for whether the chickadee should expect the light to turn on in the trial, separately for catch and non-catch trials. In both cases, coefficients corresponding to these variables were significantly non-zero (* indicates p < 0.05, calculated as in panel (a)), indicating that the early response predicted whether the light would turn on. For (c), included cells were the same as in Fig. 3h (n = 402 cells).

What accounts for the early response? We first considered that it may be a visual response to the previous fixation. Whenever a chickadee successfully gazed at a target, its preceding fixation also tended to be slightly closer to that target (median amplitude of 39° for saccade landing on the target vs. 46° for the following saccade; Extended Data Fig. 1e). Therefore, a neuron selective for gazes towards one target might also have elevated firing in response to preceding gazes. However, we found that this type of tuning did not fully explain the data. Rather, firing during saccades was independently tuned to both the previous and the next fixation (Fig. 3e). We confirmed this result using a linear mixed effects model that accounted for the distances of both the previous and the next fixation to preferred target (Extended Data Fig. 8a). Even two saccades preceded by identical gaze fixations produced different firing rates depending on the fixations that followed (Fig. 3f). We concluded that the early response is not purely visual; rather, it appears to be predictive of the upcoming gaze.

These results suggest an intriguing hypothesis: at the end of each gaze fixation, the hippocampus encodes both a prediction of what the bird is about to see and a response to what the bird just saw. In individual neurons, this pattern appears as a biphasic (early and late) response aligned to saccades towards the preferred target. Thanks to the closed-loop design of our task, we could try to separately influence these two responses. We analyzed each cell’s responses to its preferred gaze target. We compared saccades when that target site was rewarded (and the light cue turned on) to saccades when the same site was unrewarded (and the light cue stayed off; Fig. 3g). In the early response, firing rates were identical between these two conditions; this was expected because the hippocampus had no *a priori* information about the upcoming light cue. In contrast, firing rates diverged in the late response, with higher rates in the light-on condition. We concluded that late in the fixation period, the hippocampus responds to visual stimuli. This late response can represent information beyond the location of the gaze target.

Next, we asked how hippocampal responses changed if the chickadee was able to predict the upcoming light cue. Instead of rewarding a random site on each trial, we implemented a blocked-trial task in three birds, in which the same site was rewarded six trials in a row. Chickadees found the rewarded site faster during these trial blocks, suggesting that they understood the structure of the task (Extended Data Fig. 1f). We found that in the blocked-trial task, firing rates diverged during the early response (Fig. 3h; Extended Data Fig. 8b). In other words, the early response was predictive of the light cue, even before the chickadee gazed at the correct site.

After diverging in the early response, firing rates continued to separate during the late response. Here, we wanted to disambiguate the bird’s prediction from the actual reaction to the light cue. We included a small number of “catch trials” in which the reward was omitted – i.e., the chickadee expected the light to turn on, but the light actually stayed off. Absence of the expected light cue suppressed neural activity: the late response was weaker in catch trials compared to light-on trials. However, this response was still stronger than in those trials when the chickadee did not expect the light cue.

In summary, activity during each saccade represents a mixture of information about the recently completed gaze fixation and a prediction about the upcoming fixation. We are unsure of what exactly the hippocampus predicted in our blocked-trial task: it could be simply the visual stimulus, or something more complex like reward anticipation. Regardless, the important conclusion is that the hippocampus represents both internally-driven and externally-driven information about visual targets. This coding is temporally coordinated by saccadic head movements, multiple times per second.

## Inhibitory dynamics during saccades

Saccade-related dynamics that we observed are not unlike other fast phasic processes in the hippocampus – most notably the theta oscillation. Inhibition plays a major role in these processes. For example, different classes of inhibitory interneurons fire at different phases of theta^39^. Such precise timing is thought to be important for the temporal patterning of excitatory cells and for the mechanisms of synaptic plasticity^40,41^. Birds do not appear to have continuous oscillations in the hippocampal local field potential (LFP)^20^. We wondered whether their inhibitory (I) and excitatory (E) cells were instead temporally coordinated by head saccades.

**Extended Data Figure 9.**
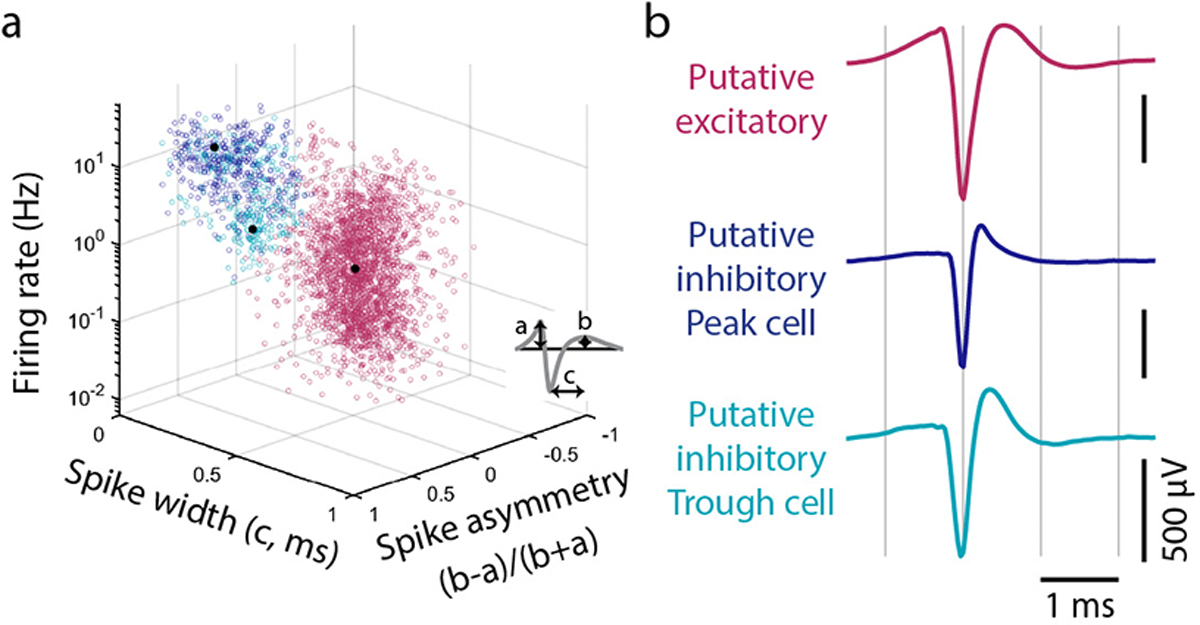
Classification of cell types in the chickadee hippocampus **(a)** All recorded units plotted according to their mean firing rate across the session and two features of spike waveforms shown in the diagram. These measurements separate putative excitatory (*pink*) from putative inhibitory (*blue* and *teal*) cells. Inhibitory cells are further classified by their responses during saccades (Fig. 4) into Peak (*blue*) and Trough (*teal*) types; these types show some systematic differences in firing rate and spike waveforms. **(b)** Average spike waveforms of three example cells, one from each of the categories shown in (a).

**Figure 4.**
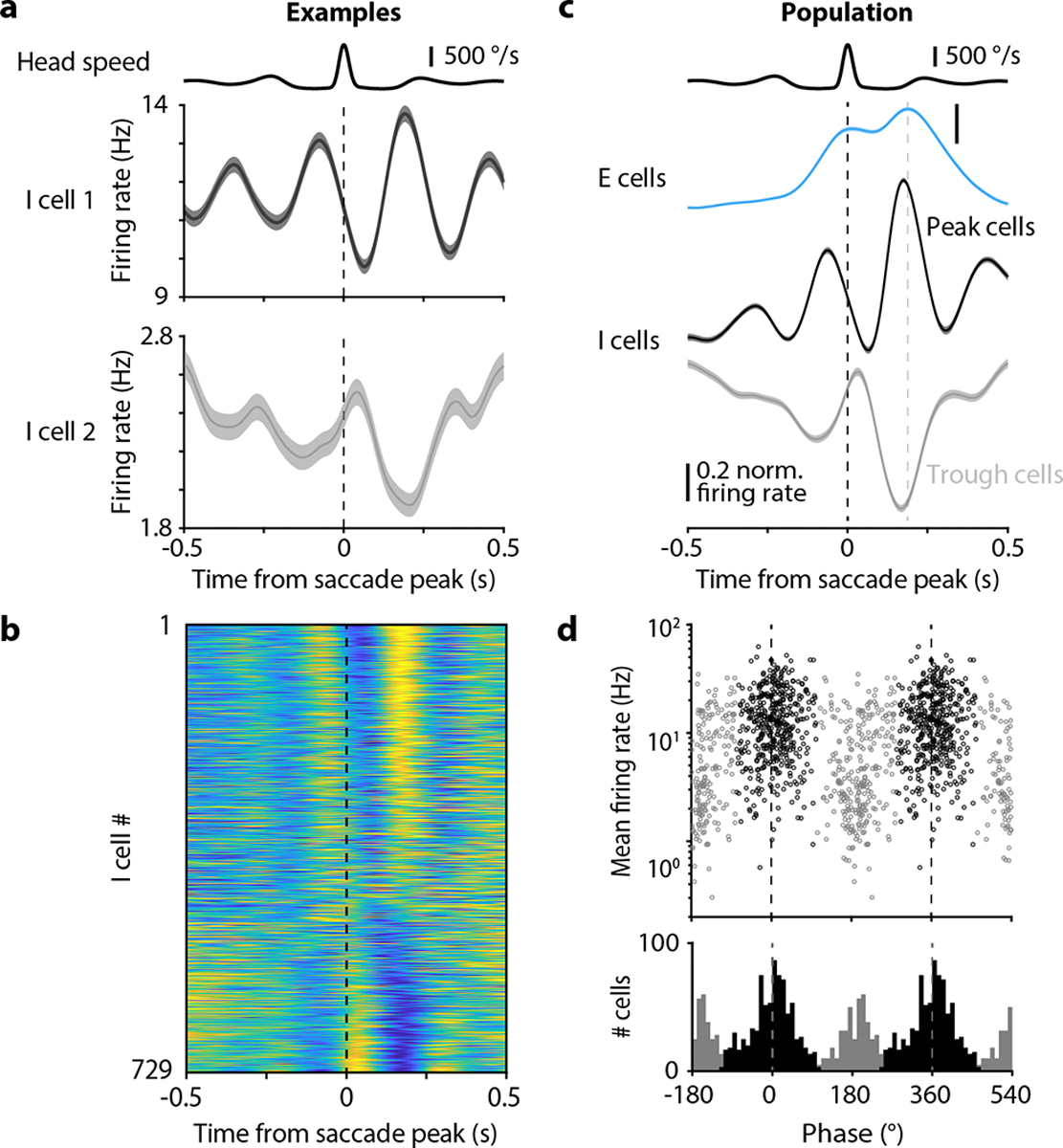
Inhibitory dynamics during saccades. **(a)** Activity of two inhibitory (I) cells, averaged across all saccades regardless of target, otherwise plotted as in Fig. 3b. **(b)** Responses of all I cells, sorted by the instantaneous Hilbert phase measured at during the late response (at 187 ms). Each cell’s response is normalized from minimum (blue) to maximum (yellow). **(c)** Responses of excitatory (E) cells and two types of I cells – Peak and Trough, classified according to the phase at 187 ms. Dotted lines indicate 0 and 187 ms – the time of the second peak in excitatory cell firing. Traces are mean ± S.E.M, averaged within a cell and then across cells. **(d)** Distribution of phases and mean firing rates (across the entire session) for all I cells. Cells show a clear bimodality across the population.

As in previous studies^41,20,21^, we classified chickadee hippocampal neurons as E or I using firing rates and spike waveforms (Extended Data Fig. 9). Up until this point, we have been reporting only the activity of E cells, but now consider I cells. Our analysis revealed two types of gaze responses in I cells. Some neurons (e.g. cell 1 in Fig. 4a) produced a smaller trough in firing shortly after the saccade and a larger peak in firing later during the fixation. Other neurons (e.g. cell 2 in Fig. 4a) instead produced a smaller peak early and a larger trough later. We summarized these patterns by measuring the instantaneous phase of each cell’s response at a fixed time after the saccade. Across the population, these phases had a clearly bimodal distribution, with two groups of cells roughly 180° apart (Fig. 4b–d). These groups (“Peak” and “Trough” cells) also had different mean firing rates and spike waveforms (Fig. 4d, Extended Data Fig. 9). We concluded that Peak and Trough cells likely correspond to different classes of hippocampal interneurons.

Because saccades followed each other in quick succession, firing rates of I cells contained multiple peaks (Fig. 4a–c). For example, Peak cells produced the largest firing peak at 180 ms after the saccade. But since the median inter-saccadic interval was ~270 ms (Fig. 1c), averaging across saccades produced multiple smaller versions of the same peak spaced by the inter-saccadic interval (e.g. at −90 and 450 ms). Owing to the multiple peaks, firing rates appeared to oscillate. Since saccades were irregularly timed, this was not a true periodic oscillation. Rather, the firing of I cells should be considered a quasiperiodic oscillation entrained by saccades. In this oscillation, Peak and Trough cells fire out-of-phase with each other, and are both phase-shifted relative to the E cells. In summary, quasiperiodic saccade-related activity in chickadees has a major feature in common with theta oscillations in rodents: subtypes of interneurons that fire at different phases relative to each other and to the excitatory population.

## DISCUSSION

We have uncovered a critical role of vision in remotely driving place representations. Previously, gaze responses have been studied extensively in the primate hippocampus^10–15^. Considered without regard for place, chickadee hippocampal activity resembles responses in monkeys that also correlate to gaze location. Some of the primate hippocampal activity is invariant to the location of the animal, which is a property we demonstrated in chickadees using our all-to-all task. The similarity between birds and primates is notable because foveal vision in these species evolved independently^4^ and relies on different contributions of head and eye movement. Localized gaze responses therefore appear to be fundamental to hippocampal function across these highly visual, but phylogenetically distant species. Prior to our work, there was a major missing link between these gaze responses and the well-studied spatial representations in the hippocampus.

The main reason for this gap is that experimental primates are usually stationary, and technical challenges discourage their recordings during movement. Only a few recent studies have managed to track gaze in navigating monkeys, either in virtual reality or with head-mounted devices^12–15^. These studies have not found the same close correspondence of place and gaze responses that we observed in chickadees. They have also reported only modest place and gaze selectivity, compared to the robust firing fields in chickadees. One issue is sampling: in these studies, monkeys rarely view and visit the same locations (most gazes are at distal landmarks rather that the floor). Another issue is that much of the monkey gazing behavior is passive, rather that deliberately directed at spatial goals. In contrast, our visual search task forced chickadees to gaze directly at their navigational targets, and ensured that these targets were behaviorally relevant. The motivational or attentional state could have a major effect on the hippocampal signals. Finally, our task design allowed cleanly separating periods of visual search from periods of navigation. Such analysis proved to be critical, since the coding of gaze and place was different during these non-overlapping periods of time. Future experimental designs and analyses might uncover more similarities between birds and primates.

Overlapping visual and spatial responses exist not only in the hippocampus, but in the visual system itself. Experiments in owls have demonstrated place coding in parts of the visual hyperpallium^42^. Similar spatial activity has been found in the primary visual cortex of mice^43^. These findings raise the question of where visual and spatial responses are computed. The hippocampal formation in both birds and mammals is strongly interconnected with the visual system^32–34^. It remains to be seen which features of neural activity arise in the hippocampus, which ones are inherited from upstream visual regions, and whether the organization of these signals is conserved across species.

Our results also relate to remote activation of place cells in rodents. Hippocampal activity can encode places that are different from the rodent’s actual location, both during rest^44^ and during active behavior^30,31^. Some of the activity during behavior might be influenced by vision. For example, when navigating rats point their heads at distant targets, remote activity correlates to head direction^45,46^. Activity in the rodent hippocampal formation even encodes the angle of the eye relative to the head^47^. However, remote activity can also represent places behind the head, or otherwise not visible to the animal, and therefore cannot be explained purely by vision^45^. A reasonable hypothesis is that vision at least partially affects hippocampal activity when rodents attend to distant visual targets. In most behaviors, testing this hypothesis is challenging because rodents lack a fovea and do not align their eyes precisely with visual targets^6,8,9^. However, in some behaviors like hunting, the precise visual target is known^9^. Recordings in these behaviors will be informative for comparison with our results. Conversely, future work in birds will determine whether their remote activity is fully determined by gaze or whether, as in rodents, it can sometimes be unrelated to vision.

Another intriguing connection of our work is to theta oscillations. Theta is important for several kinds of temporal coding in the hippocampus. In rodents, different molecular and morphological classes of interneurons fire at different phases of theta^39^. Their timing is essential for coordinating the firing of excitatory cells and for mechanisms of synaptic plasticity^40,41^. On each theta cycle, the hippocampus also switches between states dominated by external inputs and by internal connections^48^. This process might enable to hippocampus to fluctuate between different functions, such as memory storage during one phase of theta and memory recall during another phase. These theories of theta are hard to reconcile with the fact that other species lack, or at least have greatly reduced theta^17,20,49^. We demonstrate a potential solution: temporal patterns of hippocampal activity can instead be paced by irregular saccades, forming a quasiperiodic oscillation. Animals might store and recall spatial memories (including food cache memories in chickadees) during specific phases of the saccadic cycle. This fluctuation could be coordinated by specialized inhibitory cells – conceivably even cells homologous to those found in mammals.

Just like saccades synchronize hippocampal activity with incoming visual information, theta can synchronize activity with active sensory processes like whisking, sniffing, and stepping^50–52^. Saccadic modulation and theta might therefore be analogous processes, each adapted for the sensory behaviors of a particular animal species. Our results are consistent with work on the hippocampal LFP in bats, which is aperiodic^53^. They might also be consistent with primate data^17,54,55^ – though primates seem to retain some theta in addition to saccades. Monkey recordings so far have shown some differences in timing during saccades, including between E and I cells^15^.

Chickadee data allow us to formulate a general idea about hippocampal function. We suggest that hippocampal activity encodes the place that is currently relevant to the animal. For a ground-foraging rodent, this place is usually directly in front of the nose. For a highly visual animal like a bird or a primate, this place is usually at a distant visual target. In both cases, behavior forces some exceptions: rodents temporarily attend to distant targets to make navigational choices, while a moving bird might attend to its current location. As a result, both local and remote coding are present in the hippocampus, albeit in amounts that vary across species and behavioral tasks. The strength of our visual foraging task is that it required chickadees to use both types of coding, and to switch between them at experimentally well-defined moments in time. Our results suggest how the hippocampus can simultaneously perform local functions (forming a new spatial memory when storing a nut), and remote functions (recalling that memory from afar).

## METHODS

### Subjects

All animal procedures were approved by the Columbia University Institutional Animal Care and Use Committee and carried out in accordance with the US National Institutes of Health guidelines. Subjects were 9 adult black-capped chickadees (*Poecile atricapillus*) collected from several sites in New York State using federal and state scientific collection permits. Of these, 8 birds (5 male, 3 female) were used for neural recordings: 7 in the Random task, 3 in the Blocked-trial task, and 3 in the All-to-all task, with some birds used in multiple tasks. The ninth bird (male) was used for measurements of the accuracy of eye tracking. Experiments were conducted blind to sex because chickadees do not have noticeable sexual dimorphism. Sex was determined post-hoc. During experiments, birds were singly housed on a “winter” light cycle, 9h:15h light:dark. Primary wing feathers were trimmed to prevent flight.

### Head tracking and gaze estimation

To determine whether gaze could be estimated from head movements alone, and to estimate the direction of that gaze, we first conducted behavior-only calibration sessions for each bird. In these sessions the bird sat on a single perch and both head and eye movements were measured simultaneously (Fig. 1). For these sessions, we used the same behavioral arena as for the full task described below, but configured the floor with a single perch near a dish of seed fragments to encourage perching in one place.

We tracked head position using an infrared-based motion capture system (Qualisys Miqus cameras and Qualysis Track Manager software; Qualysis AB.) consisting of four specialized infrared cameras recording at 300 frames/s. The motion capture system tracked a 3D printed arrangement (“rigid body”) of 5 reflective markers (3M Scotchlite 7610 Reflective Tape) affixed to an implant on the bird’s head using neodymium magnets and a 3D printed kinematic mount (Fig. 1a). We calculated the position of the rigid body in the “**world**” reference frame anchored by known landmarks in the arena.

Eye position was tracked using a custom dual camera video-oculography system based on existing techniques that do not require cooperation of the subject for calibration^56,57^. The video-oculography system consisted of two cameras positioned 4 cm apart (Blackfly S BFS-U3–27S5M; lens Edmund Optics 59871 25mm/F1.4; visible light-blocking filter), and two infrared light sources positioned 11 cm apart (850 nm; Mouser 416-LST101H01IR0101). All cameras and light sources aimed at the bird sitting on the perch.

Before eye position could be recorded, there was a three step process to calibrate the combined head- and eye-tracking system. First, we determined the relative positions of the two cameras and their lens parameters using a checkerboard grid and the MATLAB computer vision toolbox (MATLAB, R2021a). This step defined a “**video-oculography**” reference frame centered on the optical center of the first camera. Second, we determined the positions of the infrared light sources relative to this video-oculography reference frame by imaging their reflections in a front surface mirror. The position and orientation of the mirror plane was determined using a checkerboard affixed to its mirrored surface. Finally, we aligned the world and video-oculography reference frames by determining the position of three reflective markers in both systems simultaneously.

For every frame in which the eye was visible in both video-oculography cameras, we used a published algorithm^58^ to determine the pixel coordinates of the pupil and the reflections of the two IR light sources on the corneal surface. Using our camera calibration described above, we then converted these 2D pixel coordinates to 3D positions in the video-oculography reference frame. Finally, we estimated the center of corneal curvature using the position of the two corneal reflections and the positions of the IR light sources relative to the eye and the cameras. We defined the position of the eye as the center of corneal curvature, and defined the optical axis of the eye as the vector pointing from the center of corneal curvature to the pupil center. MATLAB code for the eye tracking calibrations and analysis is available at https://github.com/hpay/eyetrack-bird). We applied several quality checks to discard frames in which the eye tracking failed.

We next defined a “**head**”-centered reference frame, which was applied to the calibration session as well as all experimental sessions. The position of the head (origin) was taken as the midpoint of the two eyes, averaged across frames. The x axis passed through the two eyes (right positive), the y axis passed through the midpoint of the two eyes and the tip of the beak (beak positive), and the z axis pointed up. We determined the orientation of the eye relative to the head in this reference frame. In Fig. 1d, we subtracted the mean horizontal and vertical angle of the eye from each data point to show the range of eye movements from rest. For the discrete visual search task described below, we used the mean position and vector of each eye in the head-centered reference frame to estimate the directions of gaze.

### Accuracy of head and eye tracking

To measure the accuracy of head tracking, we mounted the same rigid body of 5 markers that we used in experiments onto a motorized rotation stage (Physik Instrumente M-660.45). We placed the stage inside our experimental arena and rotated the rigid body in 1° increments. For each angle, we measured the average orientation of the rigid body over 330 ms of tracking data, to match a typical duration of a head saccade. We compared this orientation to the actual rotation of the motorized stage (Extended Data Fig. 2b). We also measured the translational error of each individual marker’s position, relative to the fit of all markers to the rigid body, as provided by the Qualysis Track Manager software (0.22 mm, mean residual).

To measure the accuracy of eye tracking, we instead mounted a recently euthanized chickadee onto the motorized rotation stage. We glued one of the eyelids open and kept the eye wet with saline. We placed the rotation stage in front of the video-oculography camera setup described above. We rotated the chickadee in 1° increments and used the same analyses to track the eye as we did in our calibration experiments. We compared the orientation of the eye to the actual rotation of the motorized stage (Extended Data Fig. 2c).

### Behavioral experiments

All experiments were conducted in an enclosed square arena, with a central open space 61 cm on each side, surrounded by a 2.5 cm boundary interrupted by corner posts. The walls, floor, and ceiling were black, with ~15 cm diameter bright shapes (yellow circle, pink star, blue pentagon, and green tree) positioned on each wall, centered ~30 cm above the floor. The arena was illuminated from above. White noise was played in the background to mask inadvertent room noises.

Five feeder modules were positioned in the configurations described below for each task. Each module consisted of three concentric circles: a 3D printed perch (50 mm outer diameter, 30 mm inner diameter, 6.25 mm total height above arena floor), surrounding a raised ring of LEDs (eight DotStar LEDs per ring, Adafruit Industries) mounted on a custom PCB behind a diffuser (19 mm OD, 13m mm ID, 5.25 mm height above arena floor), surrounding a motorized feeder (11.6 mm opening diameter) that dispensed tiny sunflower seed fragments (~1.5 mg each) from a cup (4 mm deep). This arrangement ensured that the bird could not see into the feeder from a different perch, given a vertical head position of 54 ± 6 mm (mean ± standard deviation, n = 58 sessions).

In the Random and Blocked-trial tasks described below, the bird was encouraged to remain on the paths between the central and outer perches by a rubber surface restricted to an X shape, with each arm 7.5 cm wide. The rest of the arena was covered by a slippery ultra-high molecular weight polyethylene surface. For the All-to-all task, the rubber surface covered the entire arena, but birds still preferred to hop directly between perches.

To motivate food consumption, birds were deprived of food for 1–3 hours from waking (at the beginning of the light-on period of the day) until the start of the experiment. Birds were weighed daily before the experiment to ensure stable weight. Sessions typically lasted 1 hour. Birds typically underwent 3–6 habituation sessions, some conducted before surgery and some afterwards. Wired electrophysiological recordings began after these sessions. Weight from the implanted recording device and the cable was partially offset by a thin strand of fiber extracted from an elastic string (Linsoir Beads, Crystal String).

The light and feeder states were controlled in real time by the animal’s behavior. We tracked behavior at 300 frames per second using the reflective head markers and the calibrated motion capture system described above. The head marker coordinates were streamed from Qualysis Track Manager to MATLAB using the software interface QTM Connect for MATLAB (Qualysis AB). Then, the saved calibrations for each bird were used to determine head position and gaze vectors for each eye. In preliminary behavioral experiments, we found that birds typically directed their gaze towards targets along a vector slightly below the optical axis of the eye (Fig. 1h, *left*). We therefore rotated the estimated gaze vectors for each eye downwards by 5° during real-time tracking. The bird’s behavior controlled the experimental flow via MATLAB code as described in detail below. Finally, MATLAB sent serial commands to an Arduino Mega to change light and feeder states.

In all tasks, seed retrieval was detected when the bird’s beak tip was within 1 cm of the central site (if light was on but feeder was closed: 8 cm; pre-training of uncalibrated birds: when the bird’s head was within 5 cm of the central site). The feeder remained open for a fixed time, T_open_. We gradually reduced T_open_ from 20 s down to 1–2 s during pre-training, with the exact value chosen such that each bird had enough time to make only one beak poke. After T_open_, the feeder closed and the light turned off. Feeder opening and closing occurred smoothly over a total duration of 1 s.

In the **Random task**, there were five identical sites arranged in an X shape. Each outer site was located 34 cm from the central site. In this task, every session started with the central site (“Center”) illuminated (“turned on”) and its feeder open. Next, one of the four outer target sites was pseudorandomly selected as the “Target”. We ensured that the same target did not occur twice in a row, and that each target was chosen a roughly equal number of times per session. During some pre-training sessions (Fig. 1g), the Target turned on after a random delay (no more than 5 s). For the remaining experimental sessions, the Target only turned on when the bird was sitting at the Center and gazing towards the Target within a threshold of 10–20° of angular deviation for at least 3 time points. The median latency from the time of peak saccade velocity to the time of light onset was 40 ms (n = 8973 trials; Fig. 3e), and the median time to fixation onset was 33 ms. Both eyes could trigger the Target to turn on. If the bird visited any outer site before triggering the Target with gaze, the Center turned on and the program waited for the bird to return to the Center. The Target turned on every time the gaze trigger was activated. The feeder actually opened with a probability chosen manually based on the bird’s behavior (50 – 100%) in order to maintain motivation and increase the number of trials.

After the Target turned on, there were two variants of what happened next. In the “stable” variant of the task, the Target stayed on until the bird visited the feeder. In the “transient” variant, the Target only remained on while gaze was fixated at the target, but turned on again when the bird approached the site (within 8 cm). For the Random task, both task variants were used and pooled, since the analyses did not depend on the state of the light after initial detection. For the Blocked-trial task, all sessions were transient. For the All-to-all task, which was harder for birds to learn, all sessions were stable.

After the Target turned on, the program waited for the bird to visit the Target and, if the feeder actually opened, retrieve a seed. Incorrect site visits were indicated by turning the Target off and the Center on, and requiring the bird to visit the Center before the Target would turn on again. When the bird retrieved a seed from the target feeder, Target turned off and the Center turned on (with a low probability of reward, 5–25%). We considered a single trial to consist of one correct outward dash toward the Target and one inward dash toward the Center. Thus, this task elicited self-paced but structured center-out visual search behavior, with many dashes and saccades towards the same four outer sites.

The Blocked-trial task (Fig. 3f) had an identical physical arrangement. In contrast to the Random task, however, the Target was not chosen randomly, but instead was repeated six trials in a row. For the first trial in a block, the Target always turned on every time the bird’s gaze was detected. To increase the number of trials in each condition, only the contralateral eye could trigger gaze in this task. (For birds that were trained on both the Random and the Blocked-trial task, the Blocked-trial task was always run after the Random task to avoid introducing any bias in eye usage in the Random task.) For the second through sixth trials in a block, two of the trials were pseudorandomly chosen to be “Catch” trials. On a Catch trial, the first time the bird’s gaze was detected towards the Target, the Target stayed off. On all subsequent gaze detections, the Target turned on. The Catch trials were balanced such that over a session, there was a nearly equal number (±1) of Catch trials at each position within a block. In this task, the median latency from peak saccade velocity to fixation onset was 33 ms, and the median latency to light onset was 40 ms, as in the Random task.

The All-to-all task (Extended Data Fig. 3) had five sites arranged in a pentagon. There was no Center site, and the Targets were chosen pseudorandomly in an all-to-all order. Sequences requiring the bird to visit three adjacent sites in a row were excluded from the pseudorandom assignment, and Target pair counts were balanced within a session. Incorrect visits were indicated by turning off the current Target and turning on the previous Target, requiring the bird to return to the previous site before activating a gaze trigger or feeder opening. Both eyes could trigger the Target to turn on.

### Electrophysiological recording

We developed a light-weight system for chronic recording during free behavior (REF Selmaan). Neural activity was recorded using a 64-channel silicon neural probe (Cambridge NeuroTech, H5 or H6 ASSY-236). A three-part 3D printed housing system secured the headstage and protected the probe.

Signals were amplified, multiplexed, and digitized at 30,000 Hz using a custom PCB containing a wire-bonded RHD2164 chip (Intan Technologies, LLC). Intan RHX Data Acquisition Software (Intan Technologies, LLC) recorded the neural data simultaneously with the time of each video frame from the head tracking system, and the times of light or feeder changes from the behavioral control system. Digital signals were transmitted from the bird to a computer interface board via a 12-conductor SPI cable (Intan technologies, LLC, C3213), passed through a motorized commutator (Doric Lenses, Inc., AERJ_24_HDMI).

To minimize degradation of neural signals over time, the probe contacts were left embedded in a silicone gel covering the brain when not in use. The probe was inserted to the desired depth 30 minutes before recording and retracted at the end of each session using an aluminum nano-drive (Cambridge NeuroTech).

The entire assembly was 1.1 g (0.1 g probe, 0.40 g headstage and connectors, 0.28 g drive, 0.32 g housing); cement added ~0.2 g more and the rigid body of reflective markers added 0.14 g more.

### Surgery

Surgery was conducted using a two-step procedure, largely as described in (Chettih et al 2024). In the first step, a dummy implant with a removable cap was affixed to the skull. The bird was allowed to recover and removable weights were gradually added. In the second step, the craniotomy was made and the probe was implanted.

During the first step, birds were anesthetized using 1.5% isoflurane in oxygen. Feathers were removed from the surgical site, and the bird was placed in a modified stereotaxic apparatus using ear bars and a beak clamp. The head was tilted such that the angle of the groove at the base of the upper mandible of the beak was 65° relative to the horizontal, corresponding to an angle of 30° between the bite bar and the horizontal. A silver ground wire (0.005” diameter) was implanted in the contralateral hemisphere, posterior and lateral to the hippocampus and 1 mm below the surface. The location of the probe craniotomy was marked on the surface of the skull: 3.02 – 4.05 mm anterior to lambda, 0.5 – 0.73 mm lateral to the midline. Most microdrives were implanted into the left hippocampus, with two microdrives implanted in the right hippocampus. The tilt of the head was adjusted to that the 3D printed base would sit flat on the skull when centered over the craniotomy. A short base unit was cemented over the planned craniotomy (RelyX Unicem, 3M). A removable 3D printed cap was attached to the base unit. After the surgery, buprenorphine (0.05 mg/kg) was injected intraperitoneally and the bird was allowed to recover for 1.5–2 weeks while weight was monitored. After at least 5 days, a 1 g weight was added to the dummy cap.

During the second step, the bird was anesthetized as above and injected intraperitoneally with dexamethasone (2 mg/kg). The cap of the dummy implant was removed and the remaining base unit and skull were cleaned with 70% ethanol. A craniotomy and durotomy were performed covering a 1×1 mm area centered on the coordinates given above. A 3D printed biocompatible resin insert with a small central slit (0.4 × 0.1 mm) was inserted into the craniotomy site so that it pressed gently on the brain surface and was cemented to the skull. A silicon probe mounted on a drive was then moved into place, tilted laterally by 10°−15° to target the medial hippocampus. After insertion of the probe was checked, the space above the craniotomy was filled with a protective layer of silicone elastomer (Dow Corning 3–4680 Silicone Gel). The probe was advanced to sit in the elastomer and the drive was cemented into place. The protective outer housing and the headstage were secured over the probe. The top surface of the outer housing contained a hole to access the drive screw, and a kinematic composed of two small neodymium magnets and 3D printed features to allow reliable re-positioning of the reflective markers during behavioral sessions.

### Histology

After completion of all experiments for each bird, the silicon probe was left in place overnight. Birds were given an overdose of ketamine and xylazine and were then perfused transcardially with saline followed by 4% formaldehyde. Brains were extracted and stored in 4% formaldehyde, then cut into 100 μm-thick coronal sections. Brain sections were stained with fluorescent DAPI. The position of each electrode relative to the boundary of the hippocampus was estimated by measuring the distance from the surface of the brain to the lateral ventricle along the electrode track. This measurement was used to exclude recorded cells that were likely outside the hippocampus.

### Spike sorting

All analyses were conducted in MATLAB unless noted. Spike sorting was conducted using Kilosort2.0^59^. Default settings were used, except that the high-pass filtering cutoff was set to 300 Hz and there was no minimum firing rate for good channels. A total of 25 sessions were manually curated in Phy (Python), including 15 in the current dataset and 10 from previous pilot experiments. During manual curation, the automatic labels were edited as needed to mark units as “good”, “mua” (multiunit activity), and “noise”. The remaining 55 sessions were automatically curated by applying several criteria. First, we calculated the spatial extent of each unit along the probe, as well as the cluster contamination rate determined by Kilosort, and excluded units that passed a threshold for each.

Second, we identified putative excitatory and inhibitory neurons by applying a Gaussian mixture model (GMM) to the following four electrophysiological characteristics (Extended Data Fig. 5): spike rate (log transformed), spike width, spike asymmetry, and derivative peak-trough ratio. Spike width was calculated as the time from the trough of the average spike waveform to the subsequent peak. Spike asymmetry was calculated as the relative height of the two positive peaks flanking the trough. Derivative peak-trough ratio was the log-transformed ratio of the peak amplitude to the trough amplitude of the waveform derivative. We fit the GMM on the manually curated sessions to classify cells into two clusters corresponding to putative excitatory and inhibitory neurons. We then applied the GMM to all sessions and excluded units that exceeded a distance threshold from either of the two clusters. A small number of neurons that were intermediate between the two clusters were labelled as unclassified neurons and were not considered further. Cells with fewer than 500 spikes were excluded (457/3115 cells). Code to run spike sorting and process results is available at: https://github.com/hpay/spikesort-hp-2025.

Inhibitory neurons were further clustered into two groups on the basis of their average saccade-aligned activity (see Main Text). Mean firing rate was 1.3 Hz, 14.0 Hz, 7.1 Hz in the putative excitatory, Late inhibitory, and Early inhibitory clusters, respectively; spike width was 0.51 ms, 0.27 ms, and 0.33 ms; and peak amplitude asymmetry was −0.04, 0.55, and 0.56.

### Behavioral analysis

The position and orientation of the head were tracked using the motion capture system as described above. Linear head speed was calculated by differentiating the x, y, and z position of the head and calculating absolute speed as $\sqrt{\Delta x^{2}+\Delta y^{2}+\Delta z^{2}}/\Delta t$. Angular head speed was calculated by measuring the angular difference in 3D orientation of the head between adjacent frames and dividing by Δt. Both linear and angular speed were then low-pass filtered with a Butterworth filter with a cutoff frequency of 25 Hz.

#### Hidden Markov Model

To label behavioral states, we implemented a Hidden Markov Model (HMM) based closely on previous work^60^. The HMM predicted whether the bird was saccading, fixating, or feeding at each of the five sites, or whether it was dashing between two pairs of sites. The HMM had a Gaussian observation model, representing the likelihood of observing the position, orientation, and speed of the bird’s head at each time point given each state. The most likely state was computed using the Viterbi algorithm. Input data consisted of linear speed, angular speed, and head position and tilt. Position was transformed to represent proximity to sites or lines between sites. Mean parameters of the Gaussian model were fit iteratively for each session, while the variance parameters were fixed. The transition probability matrix was fixed.

We refined the output of the HMM as follows. This refinement step was prompted by our observation of common misclassifications of dashes, saccades, and fixations. First, we cleaned up misclassified states. Any “saccades” immediately preceding feeding were combined with the feeding state. Any “saccades” immediately following a dash were combined with the dash state. Any “feeding” after a dash was combined with the dash. Second, we refined the endpoints of saccades and dashes. The start and stop times of each saccade were refined by applying velocity (400°/s) and acceleration (5000°/s^2^) thresholds. The stop time of each dash was also refined by applying velocity (150 mm/s) and acceleration (3000 mm/s^2^) thresholds.

Sessions with fewer than 5 dashes or 5 saccades with each eye to each outer target were excluded from further analysis. Unless otherwise noted, all analyses were conducted on saccades that were immediately followed by a fixation. We used the time of peak saccade velocity as the alignment point for all analyses (the “time of the saccade”).

#### Analysis of gaze strategies

To calculate the time course of gaze strategies shown in Fig. 2g and Extended Data Fig. 1h, a histogram of head orientations relative to the target was first calculated for each bird, and the peak density near the beak and near each eye (averaged across eyes) was taken as the ideal vector for frontal and lateral gaze, respectively. For each saccade in the sequence, we then determined the angular distance between either frontal gaze or lateral gaze with either eye and the target of the upcoming dash. Angular distances less than 20° counted as a “hit”, and the probability of a hit for either frontal or lateral gaze across all saccades was plotted.

### Neural analysis

#### Place and gaze responses

To construct place map examples (Fig. 2c), we included position data during dashes plus a ±1 s window before and after each dash. Spatial information was calculated using a range of delays between spikes and behavior, and the best offset was chosen. The arena floor was divided into 40×40 bins in which spike counts and occupancy time were calculated. The resulting matrices were each smoothed with a 11×11-point Hamming window then divided to yield mean firing rate.

The gaze maps (Fig. 2d) included data during gaze fixations made while at the Center site. Gaze from the specified eye was projected along the optical axis measured during the calibration session from that bird. A cone with a radius of 10° was projected on the floor. Spike counts and occupancy times were calculated, smoothed with a 9×9-point Hamming window, and divided to yield mean firing rate in each bin.

We quantified the degree of spatial tuning for each neuron for both place and gaze. We quantified place tuning by calculating the information about site identity conveyed by the cell’s firing during dashes towards each of the four outer sites. We calculated information according to:

$$I=\;\sum_{x}\frac{\lambda \left(x\right)}{\lambda }\log_{2}\frac{\lambda \left(x\right)}{\lambda }p(x)$$

where I is the information rate in bits/spike, x∈{1,2,3,4} is the site identity, p(x) is the probability that the bird visited site x, λ(x) is the mean firing rate in a ±1 s window centered on the end of each dash to site 𝑥, and $\lambda =\Sigma \lambda \left(x\right)p\left(x\right)$ is the overall mean firing rate across all included time periods^61^. A null distribution for each cell was calculated by shuffling the identity of the dash targets across trials and re-calculating spatial information for 200 repetitions. A neuron was considered a significant “place cell” if actual spatial information exceeded 99% of samples in the shuffled distribution. Spatial information was normalized for each cell by dividing actual spatial information by the mean of the shuffled distribution (Fig. 1g).

We applied the same procedure to quantify the degree of gaze tuning, but for spike counts within a window from −0.1 s to +0.3 s from the time of peak saccade velocity. Only saccades that landed on a target (within 20° of visual angle), and that occurred more than 0.5 s before the start of a dash, were included.

Firing rates were calculated over time by binning spikes at the frequency of the behavioral data acquisition (3.33 ms bins) and applying either a 100-ms or a 30-ms sigma Gaussian filter for dash or gaze responses, respectively.

Dash responses were summarized across the population (Fig. 2e) by calculating the average firing rate over time aligned to dashes to each of the four outer sites for each neuron. The peak of the response in a ±1 s window centered on the dash end was measured for each of the four sites. Saccade responses were summarized across the population (Fig. 2f) using a different approach, because the configuration of sites (separated by 90°) resulted in behavioral correlations between gaze with each eye to adjacent sites (angle between eyes 106 ± 5°, mean ± standard deviation, n = 8 birds). We estimated the separate contributions of ipsiversive and contraversive gaze to neural firing using a Poisson generalized linear model (GLM). Lasso regression was applied using the MATLAB function lassoglm with alpha = 1 and lambda = 0.005 to discourage overfitting. The model was given by:

$$\log\left(E\left(y|X\right)\right)=\beta^{\prime }X$$

where each element of y was a scalar $y_{i}$ containing the observed spike count within a window from −0.1 s before to +0.3 s after the time of peak saccade velocity for trial i. Each column of X contained the predictors $x_{i},$ which were given by:

$$x_{i}=e^{-\frac{\alpha^{2}}{\tau^{2}}}$$

where α was a vector representing the angular distance of each site from each eye’s gaze vector, contralateral (“C”) or ipsilateral (“I”) to the site of recording:

$$\alpha =\left[\alpha_{C}^{1}{\;\alpha }_{C}^{2}\;\alpha_{C}^{3}\;\alpha_{C}^{4}\;\alpha_{I}^{1}\;\alpha_{I}^{2}\;\alpha_{I}^{3}\;\alpha_{I}^{4}\right]$$

and τ was a length constant equal to 45°, determined by fitting the decay of neural activity across the population as a Gaussian function of distance.

For the All-to-all task, birds started each trial in a different location, so place codes for a preferred site could contaminate responses during gaze from that site to other non-preferred sites. To account for this, we subtracted baseline activity from both the dash and saccade responses in this task as follows. The neural response for dashes was calculated as the mean response in a ±0.5 s window centered on the dash end, minus the mean response from −2 s to −1 s. The neural response for saccades was calculated as the mean response in a 0 s to +0.3 s window aligned to the saccade, minus the mean response from −1 s to −0.2 s. Responses below baseline were included in the analysis, but the color plots are cropped at zero. All included cells had at least four saccades and four dashes for every source-target pair (20 total permutations).

We determined the selectivity of each cell for place or gaze at a single site by comparing responses for the preferred site to the next-most-preferred site. Responses for dash and gaze (either peak rates for dash, or model coefficients for gaze) were first normalized by dividing by the response for the preferred site. A selectivity index was then calculated:

$$I_{select}=r_{1}-r_{2}$$

where $r_{1}$ was the normalized response for the site with the largest response and $r_{2}$ was the response for the site with the second largest response.

In Fig 3c–d, cells are sorted by their difference in firing during the early and the late responses relative to head saccades. To define early and late responses, we calculated the mean firing aligned to saccades for each cell. We then averaged these responses across all excitatory cells with strong selectivity (>0.5) of both place and gaze responses for the same target, and finding the two peaks in the average response (Fig 4c, *blue*). For each cell, we then found the peak firing rate within a ±50 ms window centered around each of the two peaks. Cells were sorted by the difference in peak rate during these two windows.

#### Analysis of interneuron firing

We categorized the pattern of interneuron firing by first calculating the mean firing rate for each cell across all saccades. Interneurons had some selectivity, but were generally active for all saccade targets. We then performed a Hilbert transform on the mean firing rate, and stored the instantaneous phase of the response at 187 ms after the saccade peak (the time of peak firing in excitatory cells, Fig. 4c). We performed circular k-means clustering on the instantaneous phases to classify interneurons into two groups.

### Statistical analysis

All confidence intervals given in the text and figures are mean ± standard error of the mean unless otherwise specified.

## Extended Data

## Supplementary Material

Supplemental Video 1 Supplementary Video 1. Example of behavior and neural recording
Rendering of the chickadee’s head position and angle from an actual behavioral session. *Cones*: projected gazes of the two eyes ±10°; *ipsi* and *contra* indicate gazes of eyes that are ipsilateral and contralateral to the neural recording, which was in the left hippocampus in this bird. On each trial, one of the four outer sites is randomly chosen as the rewarded site. An indicator light (*white ring*) turns on at that site if the bird gazes there with either eye. The video shows two trials: one in which site 1 and one in which site 2 was rewarded. Between these two trials, the chickadee makes an error and dashes toward site 4. *Cyan dots*: spikes fired by a recorded neuron during gaze fixations from the central site, plotted at the location of estimated gaze. *Red dots*: spikes fired by the same neuron during navigation (i.e. dashes from the center toward outer targets), plotted at the bird’s current location. The neuron is selective for site 1: it fires whenever the bird dashes toward site 1 or gazes at site 1 with the contralateral eye. Note that the rendering of the chickadee’s head is enlarged approximately 1.6× for visualization purposes.

## Data Availability

Data and example code are available at https://doi.org/10.5061/dryad.tqjq2bw9n.
